# O‐GlcNAcase Inhibitor Improves Denervation‐Induced Muscle Atrophy in Mice

**DOI:** 10.1002/jcsm.70066

**Published:** 2025-09-12

**Authors:** Tomoyasu Suenaga, Shouji Matsushima, Tomoka Masunaga, Toru Hashimoto, Shingo Takada, Yoshizuki Fumoto, Soichiro Hata, Kohtaro Abe, Shintaro Kinugawa

**Affiliations:** ^1^ Department of Cardiovascular Medicine, Faculty of Medical Sciences Kyushu University Fukuoka Japan; ^2^ Division of Cardiovascular Medicine, Research Institute of Angiocardiology, Faculty of Medical Sciences Kyushu University Fukuoka Japan; ^3^ Department of Lifelong Sport, School of Sports Education Hokusho University Ebetsu Japan; ^4^ Department of Molecular Biology Hokkaido University Graduate School of Medicine Sapporo Japan; ^5^ Department of Cardiovascular Medicine NHO Fukuoka National Hospital Fukuoka Japan

**Keywords:** denervation‐induced muscle atrophy, O‐GlcNAcase, O‐GlcNAcylation, phosphorylation, skeletal muscle, thiamet G

## Abstract

**Background:**

Skeletal muscle atrophy occurs in various situations, such as denervation, fasting and ageing. Disruption of the balance between protein synthesis and degradation plays an important role in muscle atrophy, and impaired Akt phosphorylation is considered to be crucial in this process. The attachment of an O‐linked N‐acetylglucosamine motif (O‐GlcNAcylation), which is a post‐translational modification mediated by the hexosamine biosynthetic pathway, an alternative pathway of glycolysis, is involved in the regulation of protein function. Akt O‐GlcNAcylation interacts with Akt phosphorylation, thereby regulating its function. The purpose of this study was to clarify the role of O‐GlcNAcylation in skeletal muscle atrophy and to identify a therapeutic target for its prevention.

**Methods:**

Denervation was induced by cutting the sciatic nerve on the right leg of male C57BL/6J mice. A sham operation was performed on the left leg. Three days after the operation, the mice were divided into two groups: One group was treated with the O‐GlcNAcase inhibitor thiamet G (1 mg/kg body weight/day), and the other group was treated with vehicle. Seven days after the operation, the gastrocnemius muscle was collected and analysed. The effect of adeno‐associated virus serotype 1–mediated suppression of O‐GlcNAcase on skeletal muscle atrophy was also investigated. Finally, in C2C12 myotubes with adenovirus‐mediated overexpression of wild‐type Akt and O‐GlcNAcylation‐resistant mutant Akt (T479A), the interaction between the phosphorylation and O‐GlcNAcylation of Akt was investigated.

**Results:**

The weight of denervated gastrocnemius muscle was decreased by 35.6% (*p* < 0.05) compared with sham. Akt phosphorylation was decreased by 27.8% (*p* < 0.05), and the expression of the muscle‐specific ubiquitin ligases muscle atrophy F‐box (atrogin‐1) and muscle RING Finger‐1 (MuRF1) was increased in denervated muscle compared with sham. Akt O‐GlcNAcylation was decreased in denervated muscle compared with sham by 45.3% (*p* < 0.05), together with an 8.9‐fold increase in O‐GlcNAcase expression. Thiamet G reduced gastrocnemius muscle weight loss by 22.7% (*p* < 0.05) compared with vehicle, and this was achieved through an increase in Akt phosphorylation by 63.5% (*p* < 0.05) and decreases in atrogin‐1 and MuRF1 expression. The inhibition of O‐GlcNAcase by gene silencing also improved skeletal muscle atrophy. The overexpression of mutant Akt (T479A) showed less O‐GlcNAcase inhibition‐induced Akt phosphorylation than the overexpression of wild‐type Akt.

**Conclusions:**

O‐GlcNAcase inhibition improved denervation‐induced skeletal muscle atrophy in mice by increasing Akt O‐GlcNAcylation. O‐GlcNAcase may hence be a therapeutic target for preventing skeletal muscle atrophy.

## 1 Introduction

Skeletal muscle atrophy occurs in various situations, such as denervation, prolonged inactivity, excessive fasting, cancer cachexia, corticosteroid use, chronic diseases and aging [1]. It is characterized primarily by a reduction in muscle cell cross‐sectional area, accompanied by a decrease in muscle fibre number, changes in fibre type, decreased mitochondrial content, mitochondrial dysfunction and degeneration of the neuromuscular junction [2, 3], resulting in muscle weakness and reduced muscle endurance. These quantitative and qualitative changes of muscle lead to a low quality of life and are associated with a reduced healthy life expectancy as well as a reduced life expectancy [4]. Exercise is the only established therapy for preventing or treating skeletal muscle atrophy. However, unless it is performed based on an appropriate prescription (frequency, intensity, time and type), its effectiveness is insufficient. In addition, elderly people with multiple comorbidities often cannot perform exercise therapy. For such patients, neuromuscular electrical stimulation is known to be effective in preventing skeletal muscle atrophy as an alternative treatment to exercise therapy, but there is insufficient evidence regarding its long‐term effectiveness. Therefore, there is an urgent need to develop novel treatments for skeletal muscle atrophy.

Skeletal muscle atrophy, regardless of aetiology, is caused by a breakdown in the balance between protein synthesis and degradation [1, 5]. There are three major known proteolytic pathways in skeletal muscle: the lysosomal system, the calcium‐dependent proteolytic (calpain) system and the ubiquitin–proteasome system. Among them, the ubiquitin–proteasome system plays an important role in skeletal muscle atrophy [6], and the roles of the muscle‐specific ubiquitin ligases muscle atrophy F‐box (atrogin‐1) and muscle RING Finger‐1 (MuRF1) are particularly important. The phosphorylation of Akt is not only involved in protein synthesis but is also known to suppress the phosphorylation of forkhead box O3A (FoxO3A) and the expression of these ubiquitin ligases [5]. Thus, Akt phosphorylation plays an important role in both protein synthesis and degradation.

The attachment of an O‐linked N‐acetylglucosamine (O‐GlcNAc) motif to serine and threonine residues of proteins (O‐GlcNAcylation) is a dynamic and reversible post‐translational modification that alters protein function in response to cellular stresses, such as heat shock and hypoxia, and nutritional status. Abnormal O‐GlcNAcylation is also involved in the development of several diseases, including cancer, diabetes, neurodegenerative diseases and cardiovascular diseases [7]. Enzymes involved in O‐GlcNAcylation include glutamine‐fructose‐6‐phosphate amido‐transferase (GFAT), which is the rate‐limiting enzyme in the hexosamine biosynthesis pathway (HBP), an alternative pathway of glycolysis, and O‐GlcNAc transferase (OGT) and O‐GlcNAcase (OGA), which are involved in the addition and removal of O‐GlcNAc from proteins, respectively.

Two to five per cent of intracellular glucose enters the HBP [8], and nutrient availability regulates O‐GlcNAcylation through the HBP, with metabolism and O‐GlcNAcylation being closely associated with each other [9]. Changes in O‐GlcNAcylation in skeletal muscle are thought to play an important role in the physiological function and pathology of skeletal muscle [10]. O‐GlcNAcylation levels were increased in the skeletal muscle of rats that underwent treadmill exercise training for 6 weeks [11]. In addition, O‐GlcNAcylation in skeletal muscle has been reported to be decreased in humans and animal models with skeletal muscle atrophy [12, 13]. We thus hypothesized that skeletal muscle atrophy is closely associated with changes in O‐GlcNAcylation.

As O‐GlcNAcylation occurs at or near the site of protein phosphorylation, O‐GlcNAcylation is thought to play an important role in regulating protein phosphorylation [8]. Consistent with this idea, O‐GlcNAcylation of Akt was shown to enhance phosphorylation and activation of Akt in vascular smooth muscle cells, leading to the progression of vascular calcification [14]. We previously found that the pharmacological inhibition of GFAT suppressed increases in O‐GlcNAcylation and phosphorylation of Akt in the heart of isoproterenol‐induced cardiac hypertrophy mice, thereby suppressing cardiac hypertrophy to approximately normal level [15]. On the other hand, it has been reported that O‐GlcNAcylation at threonine 305 and threonine 312 of Akt suppresses its phosphorylation at threonine 308 in in vitro experiments [16]. Thus, there are conflicting reports regarding the interaction of these post‐translational modifications with respect to Akt activation.

The objectives of this study were to clarify the role of O‐GlcNAcylation in skeletal muscle atrophy and to identify a therapeutic approach targeting O‐GlcNAcylation for the treatment of skeletal muscle atrophy.

## 2 Methods

Details of all experiments performed in this study, the materials used and statistical analyses are provided in the Supporting Information.

## 3 Results

### 3.1 Changes in O‐GlcNAcylation Affect Protein Synthesis and Degradation in C2C12 Myotubes

**FIGURE 1:**
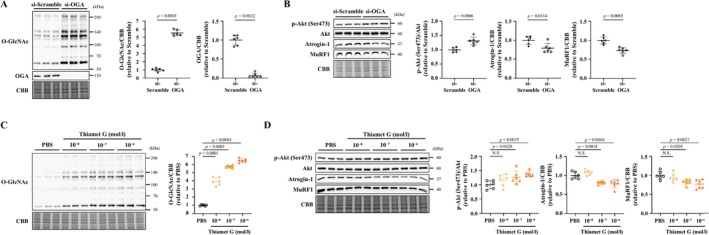
Inhibition of OGA enhances phosphorylation of Akt and decreased expression of muscle‐specific ubiquitin ligases in C2C12 myotubes. Representative western blots (left) and summary data (right) of O‐GlcNAc and OGA (A), p‐Akt (Ser473), Akt, atrogin‐1 and MuRF1 (B) levels in C2C12 myotubes transfected with si‐Scramble or si‐OGA (*n* = 6 in each group). Representative western blots (left) and summary data (right) of O‐GlcNAc (C), p‐Akt (Ser473), Akt, atrogin‐1 and MuRF1 (D) levels in C2C12 myotubes treated with PBS or different doses of thiamet G (10^−8^, 10^−7^ and 10^−6^ mol/L) (*n* = 6 in each group). p‐Akt was normalized to total Akt, and other results were normalized to non‐specific bands of the CBB‐stained gel. Data are shown as the mean ± SD. *p* values were calculated by the unpaired Student *t*‐test or Mann–Whitney *U* test for Panels A and B and one‐way ANOVA followed by the Dunnett post hoc test or Kruskal–Wallis test followed by Dunn's post hoc test for Panels C and D. Atrogin‐1, muscle atrophy F‐box; CBB, Coomassie Brilliant Blue; MuRF‐1, muscle RING Finger‐1; NS, not significant; OGA, O‐GlcNAcase; O‐GlcNAc, O‐linked N‐acetylglucosamine; p‐Akt, phosphorylated Akt; PBS, phosphate‐buffered saline; si, small interfering.

First, to investigate whether changes in protein O‐GlcNAcylation affect protein synthesis and degradation, OGA was knocked down using small interfering RNA (siRNA) against mouse OGA (si‐OGA) in C2C12 myotubes. Knockdown of OGA clearly decreased its protein levels in C2C12 myotubes and increased protein O‐GlcNAcylation (Figure 1A) and Akt phosphorylation (Ser473) (Figure 1B). On the other hand, atrogin‐1 and MuRF1 expression was decreased (Figure 1B). Knockdown of OGA significantly increased the diameter of C2C12 myotubes (Figure S1). Next, we found that the pharmacological inhibition of OGA using thiamet G or MK‐8719 increased protein O‐GlcNAcylation in a dose‐dependent manner (Figures 1C and S2A). The phosphorylation of Akt (Ser473) was increased, and atrogin‐1 and MuRF1 expression was decreased (Figures 1D and S2B) by the treatment of myotubes with these drugs at 10^−7^ mol/L. We also found that the GFAT inhibitor 6‐diazo‐5‐oxo‐L‐norleucine crystalline (DON) decreases O‐GlcNAcylation in a dose‐dependent manner (Figure S3A). The phosphorylation of Akt (Ser473) was significantly decreased at 10^−4^ mol/L, and atrogin‐1 and MuRF1 expression was increased at 10^−3^ mol/L (Figure S3B). These results showed that changes in O‐GlcNAcylation directly affect protein degradation.

### 3.2 O‐GlcNAcylation Is Reduced in Skeletal Muscle Undergoing Denervation‐Induced Atrophy

**FIGURE 2:**
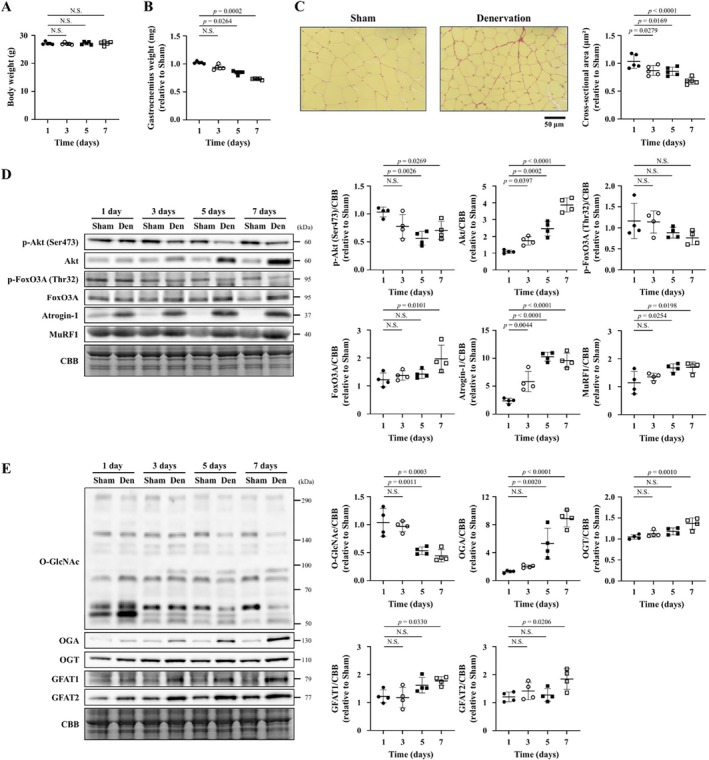
O‐GlcNAcylation is reduced in skeletal muscle undergoing denervation‐induced atrophy. Summary data of body weight (A) and gastrocnemius weight (B) of mice 1, 3, 5 and 7 days after the sham or denervation operation (*n* = 5 in each group). (C) Representative high‐magnification photomicrographs (left) of gastrocnemius muscle tissue sections stained with Picrosirius Red 7 days after the sham or denervation operation. Summary data (right) of cross‐sectional area of myocytes in the gastrocnemius muscle of mice 1, 3, 5 and 7 days after the sham or denervation operation (*n* = 5 in each group). Representative western blots (left) and summary data (right) of p‐Akt (Ser473), Akt, p‐FoxO3A (Thr32), FoxO3A, atrogin‐1 and MuRF1 (D), and O‐GlcNAc, OGA, OGT, GFAT1 and GFAT2 (E) levels in the gastrocnemius of mice 1, 3, 5 and 7 days after the sham or denervation operation (*n* = 4 in each group). Results were normalized to non‐specific bands of the CBB‐stained gel. Data are shown as the mean ± SD. *p* values were calculated by one‐way ANOVA followed by the Dunnett post hoc test or Kruskal–Wallis test followed by Dunn's post hoc test. Atrogin‐1, muscle atrophy F‐box; CBB, Coomassie Brilliant Blue; GFAT, glutamine fructose‐6‐phosphate amidotransferase; MuRF‐1, muscle RING Finger‐1; NS, not significant; OGA, O‐GlcNAcase; O‐GlcNAc, O‐linked N‐acetylglucosamine; OGT, O‐GlcNAc transferase; p‐Akt, phosphorylated Akt; p‐FoxO3A, phosphorylated forkhead box O3A.

To investigate the role of O‐GlcNAcylation in skeletal muscle atrophy, a mouse denervation‐induced skeletal muscle atrophy model created by surgical removal of the sciatic nerve was used. First, the time course of changes in the skeletal muscle of mice after denervation was evaluated. Body weight was not changed within 7 days after the operation (Figure 2A). The weight of the denervated gastrocnemius muscle was decreased in a time‐dependent manner after the operation compared with that of the sham‐operated muscle (Figure 2B). Changes in the cross‐sectional area of myocytes in gastrocnemius muscle fibres were consistent with the changes in gastrocnemius muscle weight (Figure 2C). The phosphorylation of Akt (Ser473) was significantly decreased in the denervated muscle compared with the sham‐operated muscle 5 days after the operation, whereas Akt level was increased in the denervated muscle over time (Figure 2D). It has been reported that in various skeletal muscle atrophy models, activation of FoxO3A through decreased Akt phosphorylation leads to increased expressions of atrogin‐1 and MuRF1 [17, 18]. In line with the decreased phosphorylation of Akt, phosphorylation of FoxO3A (Thr32) tended to be decreased, and FoxO3A expression was significantly increased in the denervated muscle (Figure 2D). Furthermore, expression levels of atrogin‐1 and MuRF1 were also significantly increased (Figure 2D). The phosphorylation of mammalian target of rapamycin (mTOR) (Ser2448) and p70S6 kinase (p70S6K) (Thr389) was increased in the denervated muscle (Figure S4). These changes are thought to occur as a compensation for skeletal muscle atrophy. The causes of the decreased phosphorylation of Akt have been suggested to be feedback inhibition owing to the activation of mTOR and p70S6K [19].

Protein O‐GlcNAcylation was found to decrease in the denervated muscle over time compared with the sham‐operated muscle, which was accompanied by a significant increase in the expression of OGA mRNA and protein (Figures 2E and S5). On the other hand, the expression levels of OGT, GFAT1 and GFAT2 were increased in the denervated muscle (Figure 2E). These increases were considered to be compensatory reactions in response to the decrease in protein O‐GlcNAcylation. As the substrates for O‐GlcNAcylation are produced from an accessory pathway of glycolysis, we analysed the expression of hexokinase II and pyruvate kinase M, which are key enzymes in glycolysis. However, we found no difference in their expression levels between the sham and denervated muscles (Figure S6). Taken together, our results showed that protein O‐GlcNAcylation is decreased in skeletal muscle undergoing denervation‐induced atrophy, which was mediated by an increase in OGA protein level.

### 3.3 Thiamet G Treatment Improves Denervation‐Induced Skeletal Muscle Atrophy

**FIGURE 3:**
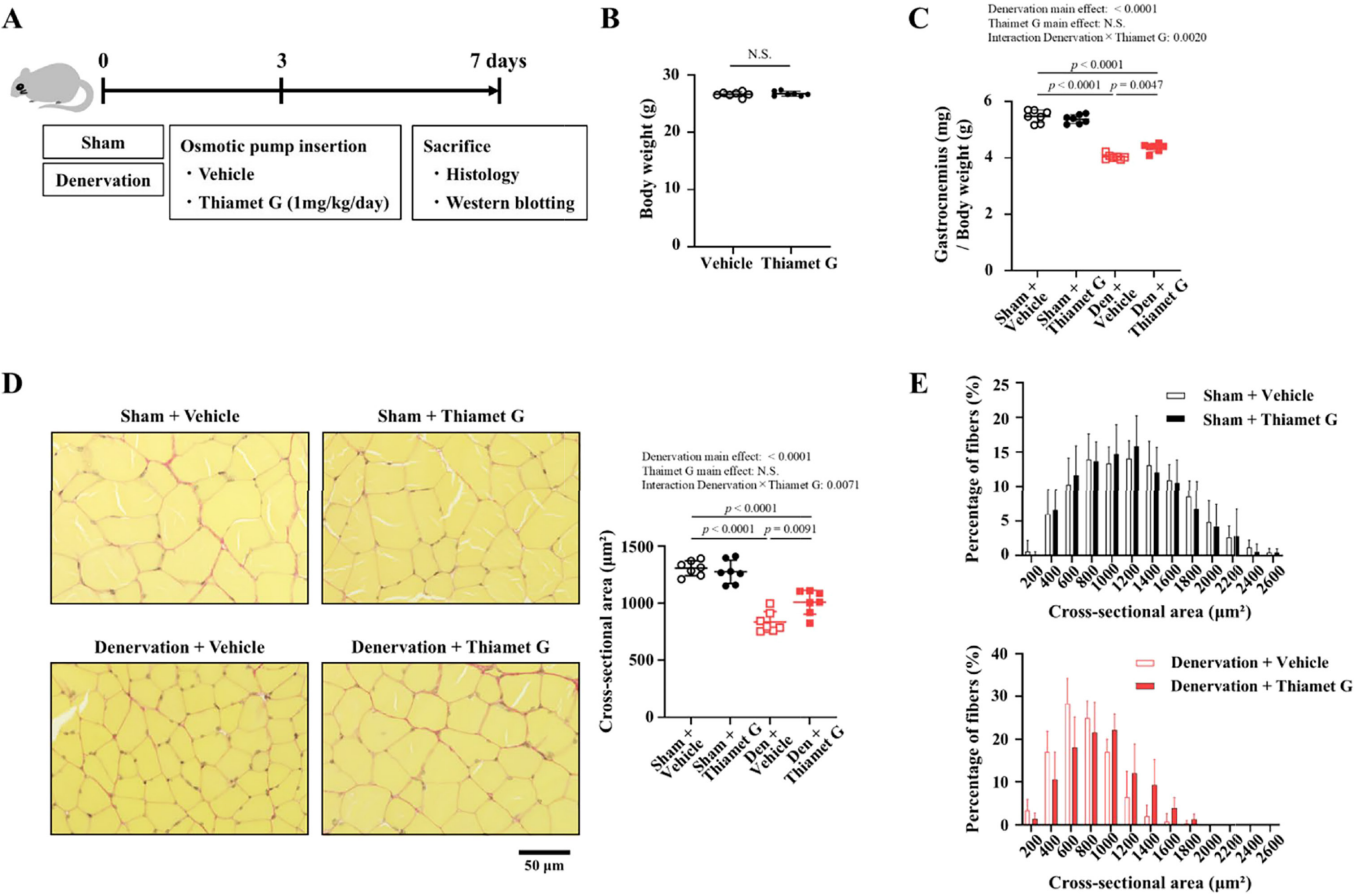
Thiamet G treatment improves denervation‐induced skeletal muscle atrophy. (A) Experimental protocol for thiamet G treatment of skeletal muscle undergoing denervation‐induced atrophy. Summary data of body weight (B) and gastrocnemius weight/body weight (C) in sham + vehicle, sham + thiamet G, denervation (Den) + vehicle and Den +thiamet G groups (*n* = 7 in each group). (D) Representative high‐magnification photomicrographs (left) and summary data (right) of the cross‐sectional area of myocytes in gastrocnemius tissue sections stained with Picrosirius Red from the four groups (*n* = 7 in each group). (E) Distribution of fibre numbers per cross‐sectional area in the gastrocnemius tissue sections of the four groups. Data are shown as the mean ± SD. *p* values were calculated by the unpaired Student *t*‐test for Panel B. *p* values of the main effect for each factor and interaction effect between two factors were calculated by two‐way ANOVA with the factors of denervation and thiamet G, and if there was an interaction effect between two factors, the Tukey post hoc test was performed. Den, denervation; NS, not significant.

We first investigated the effects of different doses of thiamet G (0.01, 0.1 and 1 mg/kg/day) on denervation‐induced skeletal muscle atrophy (Figure S7A). Treatment with thiamet G increased the weight of the denervated muscle adjusted for body weight, and the cross‐sectional areas of myocytes of the gastrocnemius muscle in a dose‐dependent manner, without altering body weight (Figure S7B–D). Next, we investigated the therapeutic effects of thiamet G on skeletal muscle atrophy 7 days after denervation operation (Figure 3A). There was no difference in body weights between mice treated with thiamet G and those treated with vehicle (Figure 3B). The weight loss in the denervated gastrocnemius muscle of mice treated with vehicle was partially and significantly attenuated in mice treated with thiamet G (Figure 3C). The same was observed in the soleus muscle (Figure S8A). Changes in the cross‐sectional area of myocytes from denervated muscle tissue treated with thiamet G were consistent with the changes in denervated gastrocnemius muscle weight (Figure 3D). In contrast to the denervated muscles, thiamet G had no effect on sham‐operated muscles (Figure 3C,D). When the proportion of fibre number per myocyte cross‐sectional area was visualized using histograms, the histogram for the denervated muscle (Figure 3E, lower panel) was found to be shifted to the left compared with the sham‐operated muscle (Figure 3E, upper panel). Furthermore, treatment with thiamet G shifted the histogram to the right in the denervated muscles (Figure 3E, lower panel). We also investigated the therapeutic effects of thiamet G on skeletal muscle atrophy 14 days after denervation operation (Figure S9A). Again, thiamet G attenuated the development of skeletal muscle atrophy (Figure S9B). Therefore, these results demonstrate that the pharmacological inhibition of OGA improves skeletal muscle atrophy.

### 3.4 Thiamet G Treatment Attenuates the Decrease in O‐GlcNAcylated Akt and Akt Activation in Skeletal Muscle Undergoing Denervation‐Induced Atrophy

**FIGURE 4:**
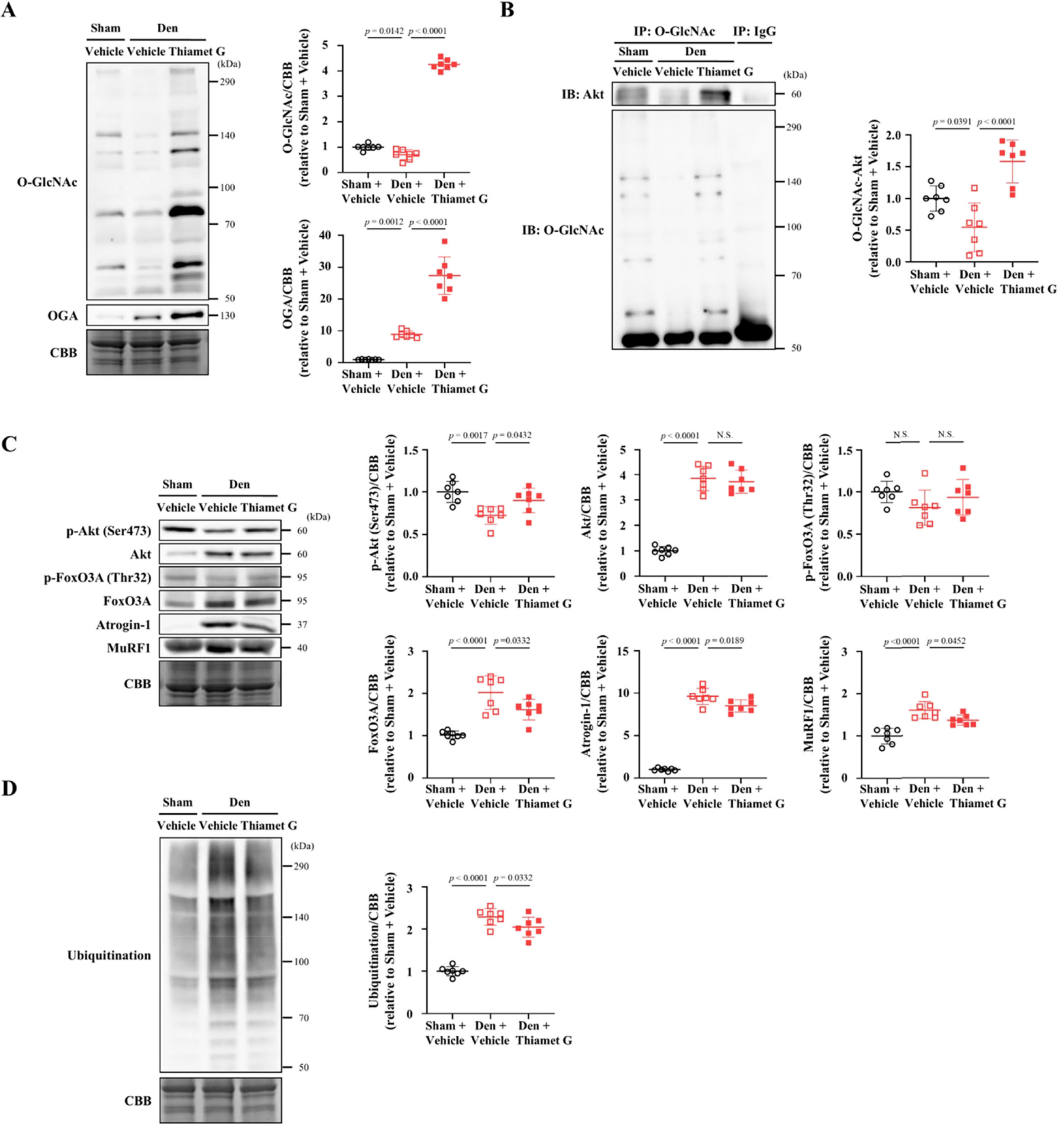
Thiamet G treatment suppresses the decrease in Akt activation and the enhancement of protein degradation in denervation‐induced skeletal muscle atrophy. (A) Representative western blots (left) and summary data (right) of O‐GlcNAc and OGA levels in gastrocnemius muscle of sham + vehicle, denervation (Den) + vehicle and Den + thiamet G groups (*n* = 7 in each group). Results were normalized to non‐specific bands of the CBB‐stained gel. (B) Immunoprecipitation assays using lysates of the gastrocnemius muscle of mice of the three groups (*n* = 7 in each group). After immunoprecipitation with control IgG or an O‐GlcNAc antibody, immunoblotting for Akt and O‐GlcNAc was performed. Representative western blots (left) and summary data (right) of O‐GlcNAc‐Akt are shown. Representative western blots (left) and summary data (right) of p‐Akt (Ser473), Akt, p‐FoxO3A (Thr32), FoxO3A, atrogin‐1 and MuRF1 (C) and ubiquitination (D) levels in the gastrocnemius muscle of mice of the three groups (*n* = 7 in each group). Results were normalized to non‐specific bands of the CBB‐stained gel. Data are shown as the mean ± SD. *p* values were calculated by one‐way ANOVA followed by the Tukey post hoc test. Atrogin‐1, muscle atrophy F‐box; CBB, Coomassie Brilliant Blue; Den, denervation; IB, immunoblotting; IP, immunoprecipitation; MuRF‐1, muscle RING Finger‐1; NS, not significant; OGA, OGlcNAcase; O‐GlcNAc, O‐linked N‐acetylglucosamine; O‐GlcNAc‐Akt, O‐GlcNAcylated Akt; p‐Akt, phosphorylated Akt; p‐FoxO3A, phosphorylated forkhead box O3A.

To investigate the mechanism mediating the anti‐atrophic effects of thiamet G in skeletal muscle undergoing denervation‐induced atrophy, proteins associated with muscle atrophy were evaluated. Protein O‐GlcNAcylation was decreased in the denervated gastrocnemius and soleus muscles, and this was increased by thiamet G treatment (Figures 4A and S8B). Although OGA was increased in the denervated gastrocnemius and soleus muscles, it was further increased in response to thiamet G treatment (Figures 4A and S8B). Our immunoprecipitation experiments demonstrated that the level of O‐GlcNAcylated Akt is significantly decreased in the denervated gastrocnemius muscle, whereas it is increased by thiamet G treatment (Figure 4B). There was no significant difference in total Akt levels between denervated gastrocnemius muscles of the vehicle‐treated and thiamet G–treated groups; however, the phosphorylation of Akt (Ser473) was significantly increased by thiamet G treatment (Figure 4C). Both insulin receptor substrate 1 (IRS1) and the phosphorylation of IRS1 (Ser 636/639) were significantly decreased in the denervated gastrocnemius muscle, but thiamet G did not affect either total IRS1 level or its phosphorylation (Ser 636/639) (Figure S10). Thus, the effects of thiamet G on Akt phosphorylation were not mediated through the well‐known upstream signal of Akt phosphorylation. Expression levels of FoxO3A, atrogin‐1 and MuRF1 were significantly decreased by thiamet G treatment compared with vehicle treatment (Figure 4C). There was no significant difference in the phosphorylation of FoxO3A (Thr32) between the thiamet G–treated and vehicle‐treated groups (Figure 4C). Activation of FoxO1 is also known to lead to an increase in the expression of various atrogenes [20]. Therefore, we evaluated FoxO1 expression and phosphorylation of FoxO1 (p‐FoxO1). Although denervation increased FoxO1 expression, p‐FoxO1 and FoxO1/p‐FoxO1, thiamet G did not affect these (Figure S11A,B). Consistent with the changes in expression of ubiquitin ligases, the increase in protein ubiquitination in the denervated gastrocnemius muscle was significantly attenuated by thiamet G treatment (Figure 4D). These results suggest that treatment with thiamet G increases O‐GlcNAcylated Akt and inhibits denervation‐induced skeletal muscle atrophy by inhibiting the decrease in Akt activation and the increase in protein degradation.

It has been reported that PGC1α expression is decreased and mitochondrial content and function are decreased in the denervation‐induced skeletal muscle atrophy model [3]. In the present study, PGC1α expression was also decreased in the denervated gastrocnemius muscles, but thiamet G did not affect it (Figure S11A,C). Furthermore, the decrease in PGC1α was associated with the activation of AMPKα and the increase in SIRT1, neither of which were affected by thiamet G (Figure S11A,C). Autophagy in skeletal muscle has been proposed to be mainly controlled by FoxO3 signalling [21]. We evaluated markers of autophagy. Expressions of p62, LC3‐I and LC3‐II were increased in denervated gastrocnemius muscle, but none of them were affected by thiamet G (Figure S11A,D).

### 3.5 Muscle‐Specific Inhibition of OGA Improves Denervation‐Induced Skeletal Muscle Atrophy

**FIGURE 5:**
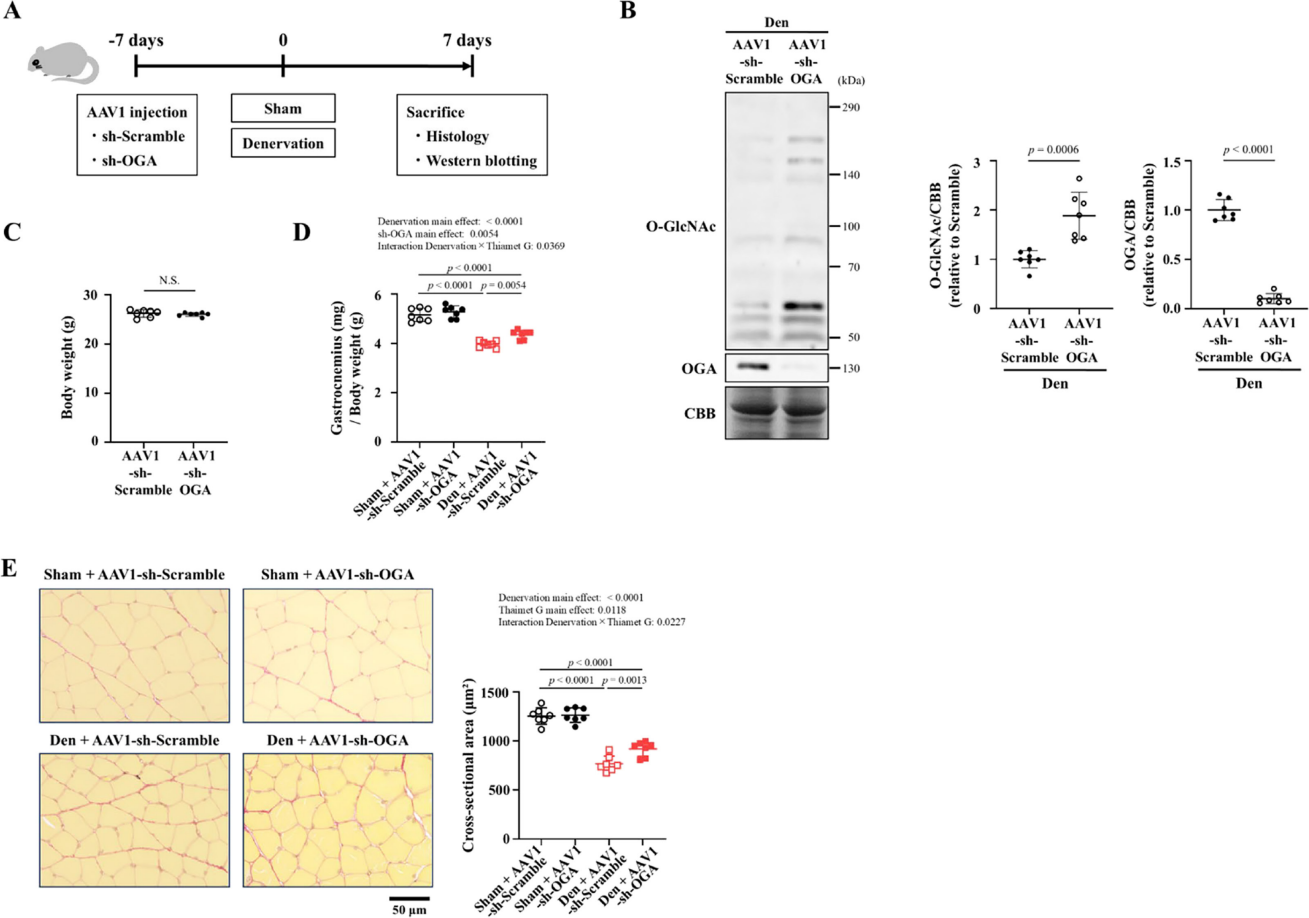
Muscle‐specific suppression of OGA improves denervation‐induced skeletal muscle atrophy. (A) Experimental protocol for AAV1‐sh‐OGA treatment of skeletal muscle undergoing denervation‐induced atrophy. (B) Representative western blots (left) and summary data (right) of O‐GlcNAc and OGA levels in gastrocnemius muscle of denervation (Den) + AAV1‐sh‐Scramble and Den + AAV1‐sh‐OGA groups (*n* = 7 in each group). Results were normalized to non‐specific bands of the CBB‐stained gel. Summary data of body weight (C) and gastrocnemius weight/body weight (D) in sham + AAV1‐sh‐Scramble, sham + AAV1‐sh‐OGA, Den + AAV1‐sh‐Scramble and Den + AAV1‐sh‐OGA groups (*n* = 7 in each group). (E) Representative high‐magnification photomicrographs (left) and summary data (right) of the cross‐sectional area of myocytes in gastrocnemius tissue sections stained with Picrosirius Red of the four groups (*n* = 7 in each group). Data are shown as the mean ± SD. *p* values were calculated by the unpaired Student *t*‐test for Panels B and C. *p* values of the main effect for each factor and interaction effect between two factors were calculated by two‐way ANOVA, with the factors denervation and AAV1‐sh‐OGA, and if there was an interaction effect between two factors, the Tukey post hoc test was performed. AAV1, adeno‐associated virus serotype 1; CBB, Coomassie Brilliant Blue; Den, denervation; NS, not significant; OGA, O‐GlcNAcase; sh, short hairpin.

To further investigate whether the muscle‐specific inhibition of OGA improves skeletal muscle atrophy, mice were treated with either adeno‐associated virus serotype 1 (AAV1)‐short hairpin RNA (sh) against OGA (AAV1‐sh‐OGA) or AAV1‐sh‐Scramble (Figure 5A). OGA expression was significantly suppressed, and protein O‐GlcNAcylation was significantly increased in mice treated with AAV1‐sh‐OGA compared with those treated with AAV1‐sh‐Scramble (Figure 5B). There was no difference in body weight between mice treated with AAV1‐sh‐OGA and those treated with AAV1‐sh‐Scramble (Figure 5C). The weight loss of the denervated gastrocnemius muscle and decrease in cross‐sectional area of myocytes from the denervated gastrocnemius muscle of mice treated with AAV1‐sh‐Scramble were partially and significantly attenuated in mice treated with AAV1‐sh‐OGA (Figure 5D,E). In contrast to the denervated muscles, AAV1‐sh‐OGA had no effect on sham‐operated muscles (Figure 5D,E). Therefore, these results demonstrate that similar to pharmacological inhibition, the muscle‐specific inhibition of OGA by gene silencing with shRNA interference ameliorates skeletal muscle atrophy.

### 3.6 Thiamet G Treatment Improves the Skeletal Muscle Atrophy and Grip Strength Reduction of Fasted Mice

Fasted mice showed a significant decrease in body weight compared with fed mice, and the weight of the gastrocnemius muscle, adjusted for tibial length, and myocyte cross‐sectional area were also significantly decreased (Figure S12A–C). The phosphorylation of Akt (Ser473) was significantly decreased in the muscle of fasted mice, without changes in Akt expression (Figure S12D). Both O‐GlcNAcylation and O‐GlcNAcylated Akt were significantly decreased in the muscle of fasted mice compared with fed mice, which was accompanied with increased OGA expression (Figure S12E,F).

**FIGURE 6:**
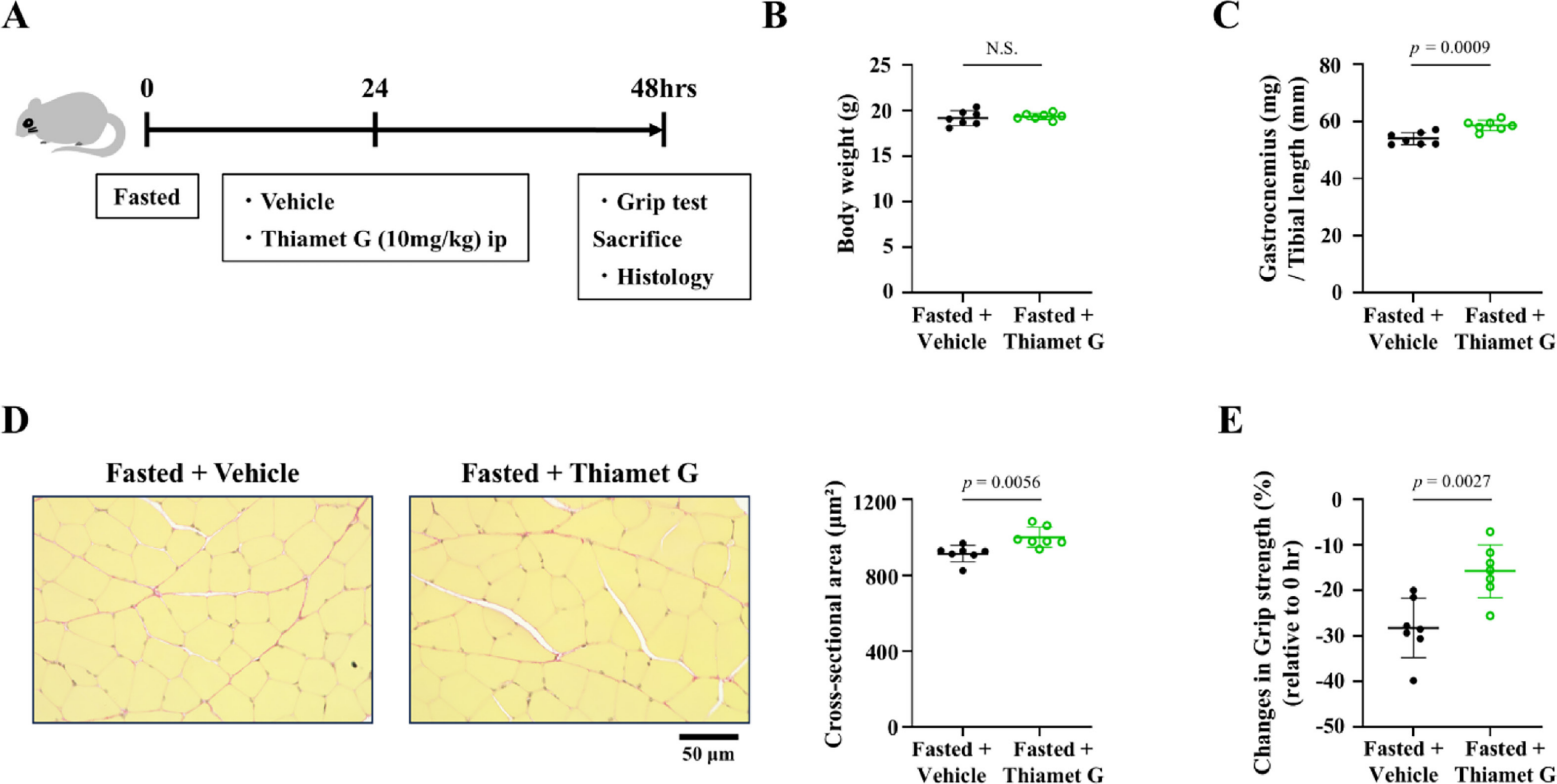
Thiamet G treatment improves fasting‐induced skeletal muscle atrophy and grip strength of mice. (A) Experimental protocol of thiamet G treatment for fasting‐induced skeletal muscle atrophy in mice. Summary data of body weight (B) and gastrocnemius weight/tibial length (C) in the fasted + vehicle and fasted + thiamet G groups (*n* = 7 in each group). (D) Representative high‐magnification photomicrographs (left) and summary data (right) of the cross‐sectional area of myocytes in gastrocnemius tissue sections stained with Picrosirius Red from the two groups (*n* = 7 in each group). (E) Changes in grip strength before (0 h) and after fasting in the two groups (*n* = 7 in each group). Data are shown as the mean ± SD. *p* values were calculated by the unpaired Student *t*‐test. NS, not significant.

Next, the therapeutic effects of thiamet G on fasting‐induced skeletal muscle atrophy were investigated (Figure 6A). There was no difference in body weight between mice treated with thiamet G and those treated with vehicle (Figure 6B). The weight of the gastrocnemius muscle adjusted for tibial length was partially and significantly increased in fasted mice treated with thiamet G compared with those treated with vehicle (Figure 6C). Changes in myocyte cross‐sectional area of the muscle tissue of fasted mice treated with thiamet G were consistent with the gastrocnemius muscle weight of fasted mice (Figure 6D). Consistent with the changes in muscle atrophy, treatment with thiamet G attenuated the reduction in grip strength of fasted mice (Figure 6E).

### 3.7 O‐GlcNAcylated Akt Is Decreased in Age‐Associated Muscle Atrophy Model Mice

The role of O‐GlcNAcylation was also evaluated in an age‐associated muscle atrophy model mice. Aged mice showed a significant increase in body weight compared with young mice, but the weight of the gastrocnemius muscle, adjusted for tibial length, and myocyte cross‐sectional area were significantly decreased (Figure S13A–C). Both total Akt and phosphorylated Akt (Ser473) were significantly decreased in the muscle of aged mice compared with young mice (Figure S13D). Although there was no overall difference in protein O‐GlcNAcylation between aged and young mice, the increase or decrease in O‐GlcNAcylation varied depending on the molecular weight of the protein (Figure S13E). As expected, O‐GlcNAcylated Akt was significantly decreased in aged mice compared with young mice (Figure S13F), which was accompanied with decreased OGT expression, but no change in OGA expression (Figure S13E). Therefore, our results suggested that some different mechanisms may be involved in the model of age‐associated atrophy, that is, sarcopenia. On the other hand, a decrease in O‐GlcNAcylated Akt may be commonly involved in various forms of skeletal muscle atrophy and may represent a general therapeutic target.

### 3.8 Interaction Between Phosphorylation of Akt at Serine 473 and O‐GlcNAcylation of Akt at Threonine 479 in C2C12 Myotubes

**FIGURE 7:**
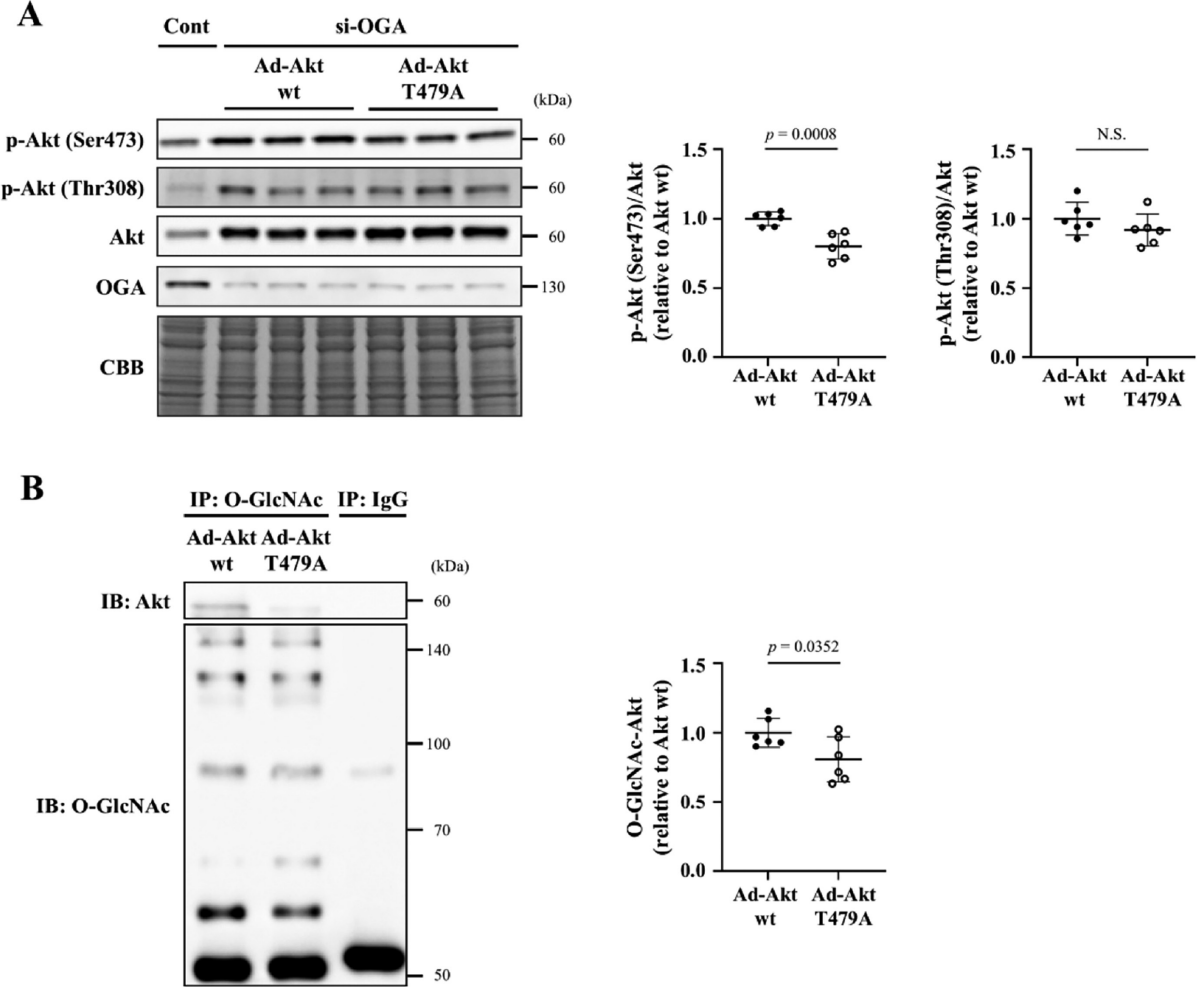
Interaction between phosphorylation of Akt at serine 473 and O‐GlcNAcylation of Akt at threonine 479 in C2C12 myotubes. (A) Representative immunoblotting (left) and summary data (right) of p‐Akt (Ser473 and Thr308) and Akt levels in C2C12 myotubes treated with an adenovirus expressing wild‐type Akt (Ad‐Akt wt) or mutant Akt (T479A) (Ad‐Akt T479A) and transfected with si‐OGA (*n* = 6 in each group). p‐Akt was normalized to total Akt. Immunoblots of the lysates of C2C12 myotubes transfected with si‐Scramble are also shown. (B) Immunoprecipitation assays using lysates of C2C12 myotubes of the two groups (*n* = 6 in each group). After immunoprecipitation with control IgG or an O‐GlcNAc antibody, immunoblotting for Akt and O‐GlcNAc was performed. Representative western blots (left) and summary data (right) of O‐GlcNAc‐Akt are shown. Data are shown as the mean ± SD. *p* values were calculated by the unpaired Student *t*‐test. ad, adenovirus; CBB, Coomassie Brilliant Blue; IB, immunoblotting; IP, immunoprecipitation; NS, not significant; OGA, O‐GlcNAcase; O‐GlcNAc, O‐linked N‐acetylglucosamine; O‐GlcNAc‐Akt, O‐GlcNAcylated Akt; p‐Akt, phosphorylated Akt; siRNA, small interfering RNA; wt, wild type.

It is known that threonine 479 and 430 of Akt are O‐GlcNAcylated, which promotes the phosphorylation of Akt at serine 473 [14]. The direct association of O‐GlcNAcylation and phosphorylation of Akt was investigated using adenoviruses expressing wild‐type Akt or O‐GlcNAcylation‐resistant mutant Akt (T479A). The overexpression of mutant Akt (T479A) resulted in a decrease in Akt phosphorylation (Ser473) induced by OGA knockdown compared with the overexpression of the wild‐type Akt (Figure 7A). On the other hand, there was no significant difference in Akt phosphorylation (Thr308) between the mutant Akt (T479A) and wild‐type Akt (Figure 7A). Akt O‐GlcNAcylation was slightly but significantly decreased in the mutant Akt (T479A) compared with wild‐type Akt (Figure 7B). Furthermore, the same experiments were performed using another O‐GlcNAcylation‐resistant mutant Akt (T430A). However, overexpression of mutant Akt (T430A) had no effect on Akt phosphorylation and Akt O‐GlcNAcylation (Figure S14). These results clearly indicated that an interaction between the phosphorylation of Akt at serine 473 and O‐GlcNAcylation of Akt at threonine 479 occurs in C2C12 myotubes.

## 4 Discussion

**FIGURE 8:**
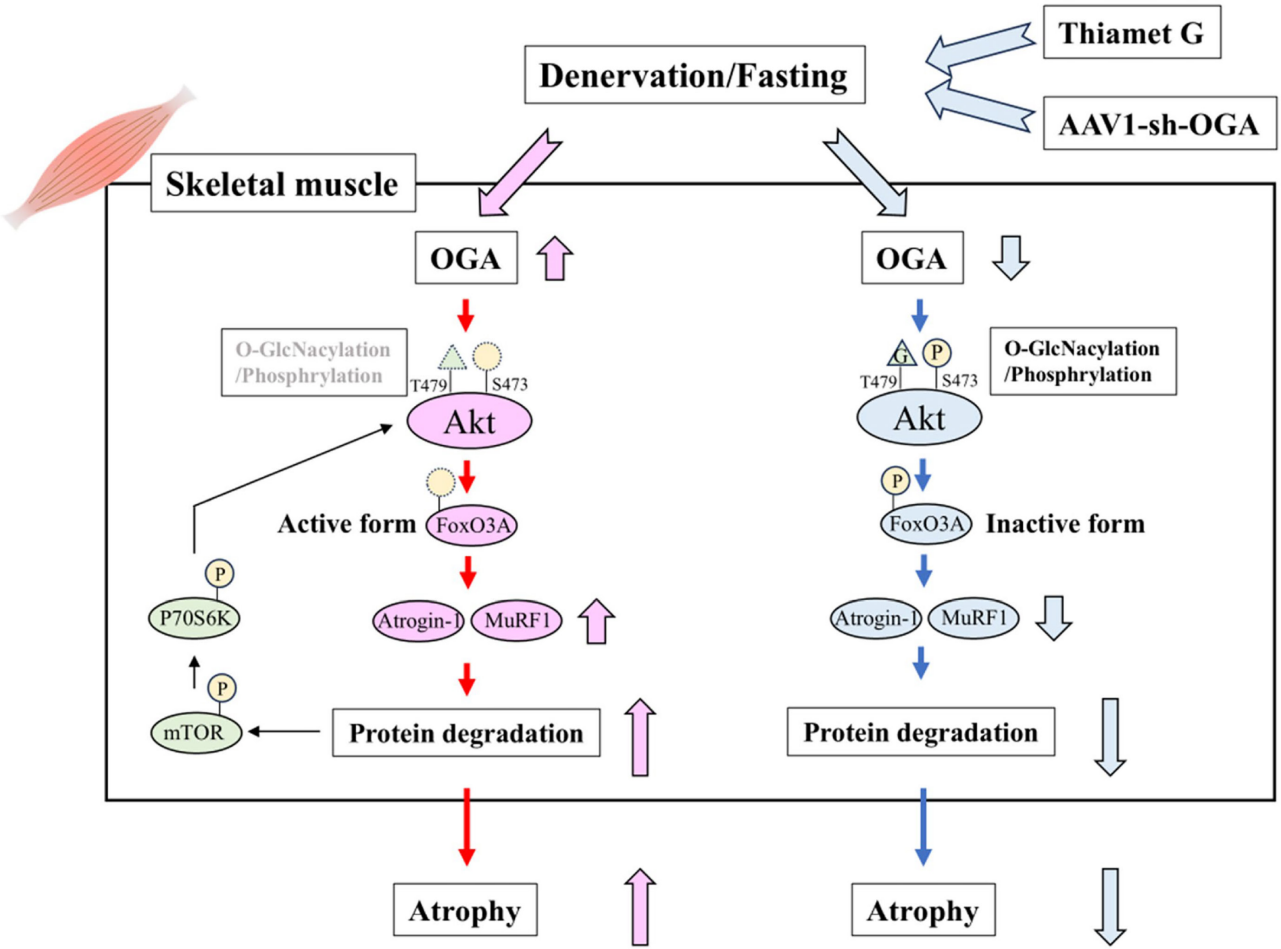
Schematic diagram of the potential roles of O‐GlcNAcylation and a possible therapeutic target for OGA inhibitors in skeletal muscle atrophy. Atrogin‐1, muscle atrophy F‐box; FoxO3A, forkhead box O3A; mTOR, mechanistic target of rapamycin; MuRF1, muscle RING Finger‐1; OGA, O‐GlcNAcase; p70S6K, p70S6 kinase.

A summary of the findings of this study is shown in Figure 8. We found that changes in O‐GlcNAcylation directly affect degradation signalling in C2C12 myotubes. Furthermore, we demonstrated that denervation‐induced atrophy reduces protein O‐GlcNAcylation in skeletal muscle, particularly O‐GlcNAcylation of Akt, and that this is mediated by an increase in OGA. O‐GlcNAcylation of Akt in skeletal muscle was found to enhance Akt phosphorylation and to play an important role in regulating the balance between protein synthesis and degradation. The inhibition of OGA by its inhibitor or by shRNA interference partially improved the denervation‐induced skeletal muscle atrophy in mice. The beneficial effect of OGA inhibition was also observed in fasting‐induced skeletal muscle atrophy. Furthermore, administration of an OGA inhibitor improved not only skeletal muscle atrophy but also the reduced muscle strength of mice. Therefore, these results suggest that OGA may be a new therapeutic target for skeletal muscle atrophy.

### 4.1 O‐GlcNAcylation in Skeletal Muscle

In the present study, we found that increasing O‐GlcNAcylation in C2C12 cultured myotubes by pharmacological and shRNA interference‐based OGA inhibition increases myotube cell diameter and suppresses proteolytic signalling (Figures 1, S1 and S2). Conversely, a decrease in O‐GlcNAcylation resulted in increased proteolytic signalling (Figure S3). Furthermore, O‐GlcNAcylation was decreased in atrophied skeletal muscle following sciatic denervation or fasting, and this was owing to increased OGA protein expression (Figures 2E and S12E). OGA activity was not evaluated in this study. It has been pointed out that OGA activity may be affected not only by quantitative changes in the OGA protein but also by its post‐translational modifications such as phosphorylation and O‐GlcNAcylation and by complex formation with OGT [22]. However, there are many unknowns regarding these mechanisms. Despite the increased expression of GFAT and OGT, O‐GlcNAcylation was decreased in atrophied skeletal muscle. In addition, O‐GlcNAcylation was increased in muscle upon treatment with thiamet G, which inhibits OGA activity. These changes are thought to have occurred in parallel with OGA activity. Previous studies reported the association between skeletal muscle atrophy and O‐GlcNAcylation. A study on skeletal muscle biopsies taken from subjects before and after 60 days of bed rest showed that O‐GlcNAcylation was decreased in the atrophied skeletal muscle after bed rest [12]. In experiments using a hindlimb unloading model of skeletal muscle atrophy, protein O‐GlcNAcylation was found to be decreased in atrophied soleus muscle [13]. This was caused by a decrease in OGT activation and an increase in OGA activity, but their protein levels remained unchanged [13]. These results support our findings in terms of changes in O‐GlcNAcylation in atrophied skeletal muscle. However, changes in the levels of O‐GlcNAcylation‐associated proteins were different between our study and these previous studies. On the other hand, in experiments using cultured C2C12 cells treated with dexamethasone, an increase in protein O‐GlcNAcylation was observed along with a decrease in muscle cell diameter [23]. This was achieved by a decrease in OGA expression, but not OGT expression. The differences between our present results and these previous results are likely owing to differences in the muscle atrophy models used. In addition, previous studies did not clarify the causal association between O‐GlcNAcylation and skeletal muscle atrophy. Most importantly, our study demonstrated that OGA inhibition improves skeletal muscle atrophy, confirming the causal association between O‐GlcNAcylation and skeletal muscle atrophy (Figures 3, 5).

### 4.2 Interaction Between Phosphorylation and O‐GlcNAcylation

The interaction between phosphorylation and O‐GlcNAcylation has received much attention to regulate signalling in response to nutrients and cellular stresses [8]. O‐GlcNAcylation of proteins occurs at serine and threonine residues, which are also potential phosphorylation sites. Several types of interactions between O‐GlcNAcylation and phosphorylation have been identified: competitive modification at the same site, competitive modification at different sites and alternating or simultaneous modification at adjacent sites [8, 16, 24, 25]. Akt is activated and exerts its function through its phosphorylation at serine 473 and threonine 308. In vascular smooth muscle cells, O‐GlcNAcylation of Akt at threonine 430 and 479 enhances its phosphorylation at serine 473, but not at threonine 308 [14]. Our previous report also showed that the O‐GlcNAcylation of Akt at threonine 479 enhances its phosphorylation at serine 473 in cardiomyocytes and that this phosphorylation is associated with cardiomyocyte hypertrophy [15]. These results are in part consistent with our present results and support the positive interaction between O‐GlcNAcylation at threonine 479 and phosphorylation at serine 473 in C2C12 cultured cells (Figure 7). On the other hand, a study using COS7 cells reported that O‐GlcNAcylation at threonine 305 and threonine 312 of Akt suppresses its phosphorylation at threonine 308 [16]. Thus, the interaction between O‐GlcNAcylation and phosphorylation is extremely complex, and it is considered difficult at present to predict how they interact on a single protein.

### 4.3 Association Between O‐GlcNAcylation of Proteins Other Than Akt and Skeletal Muscle Atrophy

The progression of skeletal muscle atrophy is associated with impaired myoblast differentiation and myotube formation. In experiments using C2C12 myoblasts, it has been reported that overall protein O‐GlcNAcylation is reduced in the early stages of myoblast differentiation and myotube formation [26, 27]. The inhibition of OGA by thiamet G or siRNA suppressed the expression of myogenin and myosin heavy chain, and inhibited myoblast differentiation and myotube formation [26]. Myogenin expression was negatively regulated by O‐GlcNAcylation of myocyte‐specific enhancer factor (MEF) 2D [27]. In the present muscle atrophy models induced by sciatic denervation or fasting, overall protein O‐GlcNAcylation was reduced (Figures 2E and S12E), which may increase myogenin expression and thus enhance skeletal muscle regeneration. Although we did not analyse changes in the levels of myogenin expression or O‐GlcNAcylation of MEF2 family proteins, the role of skeletal muscle regeneration in the progression of skeletal muscle atrophy in these models is thought to be small.

O‐GlcNAcylation may directly affect the ubiquitin–proteasome system [28]. O‐GlcNAcylation is known to suppress the ATPase activity of the 26S proteasome, which is involved in the degradation of polyubiquitinated proteins. It has been shown that the Rpt2 ATPase in the proteasome 19S cap is O‐GlcNAcylated, and its increased O‐GlcNAcylation reduces proteasome function [29]. This previous report was based on in vitro analyses, and it is unclear whether this also occurs in vivo. In addition, O‐GlcNAcylation sites are often located in proline‐, glutamic acid‐, serine‐ and threonine‐rich (PEST) sequences, which are involved in protein degradation and are targeted by the ubiquitin–proteasome pathway [30]. The activation of PEST sequences usually occurs after their phosphorylation. Owing to the competitive association between O‐GlcNAcylation and phosphorylation, O‐GlcNAcylation is thought to counter phosphorylation and protect certain substrate proteins from ubiquitin‐mediated proteolysis [31]. As mentioned above, the competitive association between O‐GlcNAcylation and phosphorylation varies among proteins. Considering that ubiquitination was found to occur throughout each of the skeletal muscle proteins analysed in our present study (Figure 4D), ubiquitination is thought to be a result of increased expression of the skeletal muscle‐specific E3 ubiquitin ligases atrogin‐1 and MuRF1, rather than owing to a direct association between O‐GlcNAcylation and ubiquitination. However, further detailed investigations are required in the future to clarify these points.

### 4.4 Regulators of OGA Expression

In this study, O‐GlcNAcylation was decreased, and OGA gene and protein levels were increased in skeletal muscle undergoing atrophy caused by sciatic denervation (Figure 2E). In addition, the protein expression levels of GFAT and OGT were also increased (Figure 2E). The decrease in O‐GlcNAcylation in this model is thought to mainly be owing to an increase in OGA, and GFAT and OGT were increased in compensation. It is known that OGT is increased at the gene expression level by various cell stress stimuli [32]. However, the regulators of OGA expression remain unknown. Changes in OGA expression are known to occur in response to changes in O‐GlcNAcylation to optimize the changes [33]. In a study using CL1–5 human lung adenocarcinoma cells, the reduction in O‐GlcNAcylation caused by treatment with the GFAT inhibitor DON was shown to reduce OGA gene and protein expression levels [34]. In this case, the reduction in OGA gene expression was attenuated by simultaneous treatment with a histone deacetylase (HDAC) inhibitor. Therefore, it was suggested that OGA gene expression is partly regulated epigenetically and that its expression may be suppressed by HDAC. It has been shown in a mouse sciatic denervation model that a reduction in HDAC9 expression is associated with an increase in atrogin‐1 and MuRF1 expression in atrophic skeletal muscle [19], and the same mechanism may be involved in the increase in OGA expression. Clarifying the mechanism behind the increased OGA expression is an important future research topic.

### 4.5 Clinical Application of OGA Inhibitors

The most important finding of this study was that the administration of an OGA inhibitor attenuated the development of denervation‐induced skeletal muscle atrophy in mice (Figure 3). The effect of the OGA inhibitor was also observed against fasting‐induced skeletal muscle atrophy (Figure 6). Furthermore, the OGA inhibitor improved not only skeletal muscle mass but also skeletal muscle function (Figure 6). Recently, OGA inhibitors have attracted attention, and several OGA inhibitors have been developed [35]. Thiamet G, which was used in this study, is a highly effective and selective OGA inhibitor designed to mimic the oxazoline intermediate of O‐GlcNAc hydrolysis and has been widely used in animal studies [24]. MK‐8719, a close analogue of thiamet G, was developed with the aim of increasing its brain penetration and is currently in phase 1 clinical trials for the treatment of progressive supranuclear palsy [36]. In addition, non‐carbohydrate small molecule OGA inhibitors with diverse backbones have been identified by high‐throughput screening and structure‐based virtual screening. In particular, the piperazine derivative ASN90 and the piperidine derivative LY3372689 are in clinical development for Alzheimer's disease, and both have progressed to Phase 2 clinical trials [37]. Thus, OGA inhibitors are drugs with high potential for clinical use and are considered to be potential treatments for skeletal muscle atrophy by targeting OGA. O‐GlcNAcylation has an impact on a variety of cellular processes, including the cell cycle, signal transduction and protein function. Therefore, OGA inhibitors may have various adverse effects, and further investigation is needed.

### Limitations of This Study

There are several limitations to this study that should be mentioned. First, in the present study, thiamet G was started 3 days after denervation, and its efficacy was observed up to 7 or 14 days. It is unclear whether the efficacy would be observed if thiamet G treatment was started at a later time point. It is known that skeletal muscle atrophy progresses in a time‐dependent manner in the sciatic denervation model [3]. Our observation up to 14 days after the operation did not capture the peak of skeletal muscle atrophy in this model. Therefore, the longer‐term therapeutic effects of thiamet G remain unknown. Analysis of the longer‐term efficacy of OGA inhibition in various skeletal muscle atrophy models including ageing or hindlimb unloading as well as the denervation‐induced muscle atrophy model will be required in the future. Second, we were unable to measure the lower limb muscle strength in the sciatic denervation model due to technical issues. Functional contractile capacities of skeletal muscle isolated from mice were not evaluated in this study. It is known that many contractile proteins in skeletal muscles are O‐GlcNAcylated, which regulates the contractile properties of skeletal muscles [38, 39]. It is possible that the suppression of grip strength loss by thiamet G (Figure 6E) is owing not only to the suppression of muscle mass loss but also to changes in the contractile properties of skeletal muscles. Considering the clinical significance, to examine the effects on functional performance or functional contractile capacities is a crucial issue that remains to be addressed in the future.

In conclusion, OGA inhibition improved denervation‐induced and fasting‐induced skeletal muscle atrophy and muscle weakness in mice by increasing O‐GlcNAcylation, particularly O‐GlcNAcylation of Akt. OGA may hence be a therapeutic target for improving skeletal muscle atrophy.

## Conflicts of Interest

The authors declare no conflicts of interest.

## Supporting information


**Table S1:** Primary and secondary antibodies used in this study.

**Figure S1:** Inhibition of OGA increases the diameter of C2C12 myotubes. Representative photographs (left) and summary data (right) of the mean diameters of C2C12 myotubes transfected with si‐Scramble or si‐OGA (*n* = 6 in each group). Data are shown as the mean ± SD. *p* values were calculated by the unpaired Student *t*‐test. OGA, O‐GlcNAcase; si, small interfering.

**Figure S2:** MK‐8719 enhances the phosphorylation of Akt and decreases the expression of muscle‐specific ubiquitin ligases in C2C12 myotubes. Representative western blots (left) and summary data (right) of O‐GlcNAc (A), p‐Akt (Ser473), Akt, atrogin‐1 and MuRF1 (B) levels in C2C12 myotubes treated with PBS or different doses of MK‐8719 (10^−8^, 10^−7^ and 10^−6^ mol/L) (*n* = 6 in each group). p‐Akt was normalized to total Akt, and the other results were normalized to non‐specific bands of the CBB‐stained gel. Data are shown as the mean ± SD. *p* values were calculated by one‐way ANOVA, followed by the Dunnett post hoc test. Atrogin‐1, muscle atrophy F‐box; CBB, Coomassie Brilliant Blue; MuRF‐1, muscle RING Finger‐1; NS, not significant; O‐GlcNAc, O‐linked N‐acetylglucosamine; p‐Akt, phosphorylated Akt; PBS, phosphate‐buffered saline.

**Figure S3:** DON attenuates the phosphorylation of Akt and increases the expression of muscle‐specific ubiquitin ligases in C2C12 myotubes. Representative western blots (left) and summary data (right) of O‐GlcNAc (A), p‐Akt (Ser473), Akt, atrogin‐1 and MuRF1 (B) levels in C2C12 myotubes treated with PBS or different doses of DON (10^−5^, 10^−4^ and 10^−3^ mol/L) (*n* = 6 in each group). p‐Akt was normalized to total Akt, and the other results were normalized to non‐specific bands of 13 the CBB‐stained gel. Data are shown as the mean ± SD. *p* values were calculated by one‐way ANOVA, followed by the Dunnett post hoc test or the Kruskal–Wallis test, followed by Dunn's post hoc test. Atrogin‐1, muscle atrophy F‐box; CBB, Coomassie Brilliant Blue; DON, diazo‐5‐oxo‐L‐norleucine; MuRF‐1, muscle RING Finger‐1; NS, not significant; O‐GlcNAc, O‐linked N‐acetylglucosamine; p‐Akt, phosphorylated Akt; PBS, phosphate‐buffered saline.

**Figure S4:** Changes in protein synthesis signalling in muscles undergoing denervation‐induced atrophy. Representative western blots (left) and summary data (right) of p‐mTOR (Ser2448), mTOR, p‐p70S6K (Thr389) and p70S6K levels in the gastrocnemius muscle of mice 1, 3, 5 and 7 days after denervation or sham operation (*n* = 4 in each group). Results were normalized to non‐specific bands of the CBB‐stained gel. Data are shown as the mean ± SD. *p* values were calculated by one‐way ANOVA, followed by the Dunnett post hoc test. CBB, Coomassie Brilliant Blue; Den, denervation; NS, not significant; p‐p70S6K, phosphorylated p70 ribosomal S6 kinase; p‐mTOR, phosphorylated mammalian target of rapamycin.

**Figure S5:** Gene expression of O‐GlcNAcase is increased in denervated gastrocnemius muscle Summary data of the gene expression of *OGA* in the gastrocnemius muscle of sham or denervation (*n* = 5 in each group). Gene expression was normalized to *ActB* gene expression. Data are shown as the mean ± SD. *p* values were calculated by the Mann–Whitney *U* test. OGA, O‐GlcNAcase.

**Figure S6:** Glycolytic enzyme levels do not change in skeletal muscle undergoing denervation‐induced atrophy. Representative western blots (left) and summary data (right) of hexokinase II and pyruvate kinase M levels in the gastrocnemius muscle of mice 1, 3, 5 and 7 days after sham or denervation operation (*n* = 4 in each group). Results were normalized to non‐specific bands of the CBB‐stained gel. Data are shown as the mean ± SD *p*‐values were calculated by one‐way ANOVA, followed by the Dunnett post hoc test, or the Kruskal–Wallis test followed by Dunn's post hoc test. CBB, Coomassie Brilliant Blue; Den, denervation; NS, not significant.

**Figure S7:** Thiamet G treatment improves denervation‐induced skeletal muscle atrophy in a dose‐dependent manner. (A) Experimental protocol for thiamet G treatment at different doses in mice with denervation‐induced skeletal muscle atrophy. Summary data of body weight (B), gastrocnemius weight/body weight (C) and cross‐sectional area of myocytes in gastrocnemius tissue sections (D) of mice treated with vehicle or thiamet G at different doses (0.01, 0.1 and 1 mg/kg/day) (*n* = 5 in each group). Data are shown as the mean ± SD. *p* values were calculated by one‐way ANOVA followed by the Dunnett post hoc test, or Kruskal–Wallis test followed by Dunn's post hoc test. NS, not significant.

**Figure S8:** Thiamet G treatment improves denervation‐induced atrophy of soleus. (A) Summary data of soleus weight/body weight in sham + vehicle, sham + thiamet G, denervation (Den) + vehicle and Den + thiamet G groups (*n* = 7 in each group). (B) Representative western blots (left) and summary data (right) of O‐GlcNAc and OGA levels in soleus muscle of sham + vehicle, denervation (Den) + vehicle and Den + thiamet G groups (*n* = 7 in each group). Results were normalized to non‐specific bands of the CBB‐stained gel. Data are shown as the mean ± SD. In Panel A, *p* values of the main effect for each factor and interaction effect between two factors were calculated by two‐way ANOVA with the factors of denervation and thiamet G, and if there was an interaction effect between two factors, the Tukey post hoc test was performed. In Panel B, *p* values were calculated by one‐way ANOVA followed by the Tukey post hoc test. CBB, Coomassie Brilliant Blue; Den, denervation; OGA, OGlcNAcase; O‐GlcNAc, O‐linked N‐acetylglucosamine.

**Figure S9:** Thiamet G treatment for 2 weeks improves denervation‐induced skeletal muscle atrophy. (A) Experimental protocol for thiamet G treatment of skeletal muscle undergoing denervation‐induced atrophy. (B) Summary data of gastrocnemius weight/body weight (C) in sham + vehicle, sham + thiamet G, denervation (Den) + vehicle and Den + thiamet G groups (*n* = 6 in each group). *p* values of the main effect for each factor and interaction effect between two factors were calculated by two‐way ANOVA with the factors of denervation and thiamet G, and if there was an interaction effect between two factors, the Tukey post hoc test was performed. Den, denervation.

**Figure S10:** Insulin receptor substrate 1 (IRS1) level is not affected by thiamet G treatment. Representative western blots (left) and summary data (right) of IRS1and p‐IRS1 (Ser636/639) levels in the gastrocnemius muscle of sham + vehicle, denervation (Den) + vehicle and Den + thiamet G (*n* = 7 in each group). Results were normalized to non‐specific bands of the CBB‐stained gel. Data are shown as the mean ± SD. *p* values were calculated by one‐way ANOVA followed by the Tukey post hoc test or Kruskal–Wallis test, followed by Dunn's post hoc test. Den, denervation; p‐IRS1, phosphorylated insulin receptor substrate 1; CBB, Coomassie Brilliant Blue; N.S., not significant.

**Figure S11:** Expression of proteins associated with FoxO1, mitochondrial biogenesis and autophagy. Representative western blots (A) and summary data (B) of p‐FoxO1, FoxO1 and FoxO1/p‐FoxO1, (C) PGC‐1α, p‐AMPKα/AMPKα and Sirt1, (D) p62, LC3‐I and LC3‐II in the gastrocnemius muscle of sham + vehicle (*n* = 7), denervation (Den) + vehicle (*n* = 7) and Den + thiamet G (*n* = 7). The blots were normalized to the non‐specific bands of CBB‐stained gel. Data are shown as the mean ± SD. *p* values were calculated by one‐way ANOVA, followed by the Tukey post hoc test. CBB, Coomassie Brilliant Blue; Den, denervation; LC3, light chain 3; p‐AMPKα, phosphorylated AMP‐activated protein kinase α; p‐FoxO1, phosphorylated forkhead box O1; PGC‐1α, peroxisome proliferator‐activated receptor γ coactivator‐1 α; Sirt1, sirtuin 1.

**Figure S12:** O‐GlcNAcylation of Akt is reduced in fasting‐induced muscle atrophy. Summary data of body weight (A) and gastrocnemius weight/tibial length (B) in fed and fasted mice (*n* = 6 in each group). (C) Representative high‐magnification photomicrographs (left) and summary data (right) of gastrocnemius tissue sections stained with Picrosirius Red of the two groups (*n* = 6 in each group). Representative western blots (left) and summary data (right) of p‐Akt (Ser473) and Akt (D) and O‐GlcNAc and OGA (E) levels of the two groups (*n* = 6 in each group). Results were normalized to non‐specific bands of the CBB‐stained gel. (F) Immunoprecipitation assays using gastrocnemius muscle lysates from the two groups (*n* = 6 in each group). After immunoprecipitation with control IgG or an O‐GlcNAc antibody, immunoblotting for Akt and O‐GlcNAc was performed. Representative western blots (left) and summary of the data (right) of O‐GlcNAc‐Akt are shown. Data are shown as the mean ± SD. *p* values were calculated by the unpaired Student *t*‐test. IB, immunoblotting; IP, immunoprecipitation; NS, not significant; O‐GlcNAc, O‐linked N‐acetylglucosamine; O‐GlcNAc‐Akt, O‐GlcNAcylated Akt; p‐Akt, phosphorylated Akt.

**Figure S13:** O‐GlcNAcylation of Akt is reduced in age‐associated muscle atrophy. Summary data of body weight (A) and gastrocnemius weight/tibial length (B) in young and aged mice (*n* = 5 in each group). (C) Representative high‐magnification photomicrographs (left) and summary data (right) of the cross‐sectional area of myocytes in gastrocnemius tissue sections stained with Picrosirius Red of the two groups (*n* = 6 in each group). Representative western blots (top) and summary data (bottom) of p‐Akt (Ser473) and Akt (D) in the two groups (*n* = 6 in each group). Representative western blots (left) and summary data (right) of O‐GlcNAc, OGT and OGA (E) levels in the two groups (*n* = 6, 3, 3 in each group). Results were normalized to non‐specific bands of the CBB‐stained gel. (F) Immunoprecipitation assays using gastrocnemius lysates of in the two groups (*n* = 6 in each group). After immunoprecipitation with control IgG or an O‐GlcNAcylation antibody, immunoblotting for Akt and O‐GlcNAc was performed. Representative western blots (left) and summary data (right) of O‐GlcNAc‐Akt are shown. Data are shown as the mean ± SD. *p* values were calculated by the unpaired Student *t*‐test. IB, immunoblotting; IP, immunoprecipitation; N.S., not significant; O‐GlcNAc, O‐linked N‐acetylglucosamine; O‐GlcNAc‐Akt, O‐GlcNAcylated Akt; p‐Akt, phosphorylated Akt.

**Figure S14:** No interaction between phosphorylation at serine 473 and O‐GlcNAcylation of Akt at threonine 430 in C2C12 myotubes. (A) Representative immunoblotting (left) and summary data (right) of p‐Akt (Ser473 and Thr308) and Akt levels in C2C12 myotubes treated with an adenovirus expressing wild‐type Akt (Ad‐Akt wt) or mutant Akt (T430A) (Ad‐Akt T430A), transfected with a small‐interfering RNA against O‐GlcNAcase (si‐OGA) (*n* = 6 in each group). p‐Akt was normalized to total Akt. Immunoblots in C2C12 myotubes transfected with si‐Scramble were also shown. (B) Immunoprecipitation assays using lysates of C2C12 myotubes in two groups (*n* = 6 in each group). After immunoprecipitation with control IgG or an O‐GlcNAc antibody, immunoblotting for Akt and O‐GlcNAc was performed. Representative western blots (left) and summary data (right) of O‐GlcNAc‐Akt are shown. Data are shown as the mean ± SD. *p* values were calculated by the unpaired Student *t*‐test or Mann–Whitney *U* test. ad, adenovirus; CBB, Coomassie Brilliant Blue; IB, immunoblotting; IP, immunoprecipitation; NS, not significant; OGA, O‐GlcNAcase; O‐GlcNAc, O‐linked N‐acetylglucosamine; O‐GlcNAc‐Akt, O‐GlcNAcylated Akt; p‐Akt, phosphorylated Akt; siRNA, small interfering RNA; wt, wild type.

## References

[1] J. T. Ehmsen and A. Hoke , “Cellular and Molecular Features of Neurogenic Skeletal Muscle Atrophy,” Experimental Neurology 331 (2020): 113379. 32533969 10.1016/j.expneurol.2020.113379

[2] S. L. Rowan , K. Rygiel , F. M. Purves‐Smith , N. M. Solbak , D. M. Turnbull , and R. T. Hepple , “Denervation Causes Fiber Atrophy and Myosin Heavy Chain Co‐Expression in Senescent Skeletal Muscle,” PLoS ONE 7 (2012): e29082. 22235261 10.1371/journal.pone.0029082 PMC3250397

[3] P. J. Adhihetty , M. F. O'Leary , B. Chabi , K. L. Wicks , and D. A. Hood , “Effect of Denervation on Mitochondrially Mediated Apoptosis in Skeletal Muscle,” Journal of Applied Physiology (1985) 102 (2007): 1143–1151. 10.1152/japplphysiol.00768.2006 17122379

[4] E. Shorter , V. Engman , and J. T. Lanner , “Cancer‐Associated Muscle Weakness – From Triggers to Molecular Mechanisms,” Molecular Aspects of Medicine 97 (2024): 101260. 38457901 10.1016/j.mam.2024.101260

[5] R. Sartori , V. Romanello , and M. Sandri , “Mechanisms of Muscle Atrophy and Hypertrophy: Implications in Health and Disease,” Nature Communications 12, no. 1 (2021): 330. 10.1038/s41467-020-20123-1 PMC7803748 33436614

[6] W. E. Mitch and A. L. Goldberg , “Mechanisms of Muscle Wasting. The Role of the Ubiquitin‐Proteasome Pathway,” New England Journal of Medicine 335 (1996): 1897–1905. 8948566 10.1056/NEJM199612193352507

[7] L. Ye , W. Ding , D. Xiao , et al., “O‐GlcNAcylation: Cellular Physiology and Therapeutic Target for Human Diseases,” MedComm (London) (2020) 4, no. 6 (2023): e456. 10.1002/mco2.456 PMC10728774 38116061

[8] C. Butkinaree , K. Park , and G. W. Hart , “O‐Linked β‐*N*‐Acetylglucosamine (O‐GlcNAc): Extensive Crosstalk With Phosphorylation to Regulate Signaling and Transcription in Response to Nutrients and Stress,” Biochimica et Biophysica Acta 1800, no. 2 (2010): 96–106. 19647786 10.1016/j.bbagen.2009.07.018 PMC2815129

[9] J. L. E. Walgren , T. S. Vincent , K. L. Schey , and M. G. Buse , “High Glucose and Insulin Promote O‐GlcNAc Modification of Proteins, Including α‐Tubulin,” American Journal of Physiology. Endocrinology and Metabolism 284, no. 2 (2003): E424–E434. 12397027 10.1152/ajpendo.00382.2002

[10] M. Lambert , B. Bastide , and C. Cieniewski‐Bernard , “Involvement of O‐GlcNAcylation in the Skeletal Muscle Physiology and Physiopathology: Focus on Muscle Metabolism,” Frontiers in Endocrinology 9 (2018): 578. 30459708 10.3389/fendo.2018.00578 PMC6232757

[11] K. H. Hortemo , P. K. Lunde , J. H. Anonsen , et al., “Exercise Training Increases Protein O‐GlcNAcylation in Rat Skeletal Muscle,” Physiological Reports 4, no. 18 (2016): e12896. 27664189 10.14814/phy2.12896 PMC5037911

[12] Y. Mounier , V. Tiffreau , V. Montel , B. Bastide , and L. Stevens , “Phenotypical Transitions and Ca^2+^ Activation Properties in Human Muscle Fibers: Effects of a 60‐Day Bed Rest and Countermeasures,” Journal of Applied Physiology (1985) 106, no. 4 (2009): 1086–1099. 10.1152/japplphysiol.90695.2008 19196916

[13] C. Cieniewski‐Bernard , Y. Mounier , J. C. Michalski , and B. Bastide , “O‐GlcNAc Level Variations Are Associated With the Development of Skeletal Muscle Atrophy,” Journal of Applied Physiology (1985) 100, no. 5 (2006): 1499–1505. 10.1152/japplphysiol.00865.2005 16357072

[14] J. M. Heath , Y. Sun , K. Yuan , et al., “Activation of AKT by O‐Linked N‐Acetylglucosamine Induces Vascular Calcification in Diabetes Mellitus,” Circulation Research 114, no. 7 (2014): 1094–1102. 24526702 10.1161/CIRCRESAHA.114.302968 PMC4030422

[15] A. Ishikita , S. Matsushima , S. Ikeda , et al., “GFAT2 Mediates Cardiac Hypertrophy Through HBP‐O‐GlcNAcylation‐Akt Pathway,” iScience 24, no. 12 (2021): 103517. 34934932 10.1016/j.isci.2021.103517 PMC8661546

[16] S. Wang , X. Huang , D. Sun , et al., “Extensive Crosstalk Between O‐GlcNAcylation and Phosphorylation Regulates Akt Signaling,” PLoS ONE 7, no. 5 (2012): e37427. 22629392 10.1371/journal.pone.0037427 PMC3358304

[17] M. Sandri , C. Sandri , A. Gilbert , et al., “Foxo Transcription Factors Induce the Atrophy‐Related Ubiquitin Ligase Atrogin‐1 and Cause Skeletal Muscle Atrophy,” Cell 117 (2004): 399–412. 15109499 10.1016/s0092-8674(04)00400-3 PMC3619734

[18] Y. Liu , T. Zhou , Q. Wang , et al., “m^6^ A Demethylase ALKBH5 Drives Denervation‐Induced Muscle Atrophy by Targeting HDAC4 to Activate FoxO3 Signalling,” Journal of Cachexia, Sarcopenia and Muscle 13 (2022): 1210–1223. 35142084 10.1002/jcsm.12929 PMC8978003

[19] L. A. Tintignac , H. R. Brenner , and M. A. Ruegg , “Mechanisms Regulating Neuromuscular Junction Development and Function and Causes of Muscle Wasting,” Physiological Reviews 95, no. 3 (2015): 809–852. 26109340 10.1152/physrev.00033.2014

[20] G. Milan , V. Romanello , F. Pescatore , et al., “Regulation of Autophagy and the Ubiquitin‐Proteasome System by the FoxO Transcriptional Network During Muscle Atorphy,” Nature Communications 6 (2015): 6670. 10.1038/ncomms7670 PMC4403316 25858807

[21] C. Mammucari , G. Milan , V. Romanello , et al., “FoxO3 Controls Autophagy in Skeletal Muscle In Vivo,” Cell Metabolism 6 (2007): 458–471. 18054315 10.1016/j.cmet.2007.11.001

[22] A. K. Nagel and L. E. Ball , “O‐GlcNAc Transferase and O‐GlcNAcase: Achieving Target Substrate Specificity,” Amino Acids 46, no. 10 (2014): 2305–2316. 25173736 10.1007/s00726-014-1827-7 PMC4584397

[23] L. Massaccesi , G. Goi , C. Tringali , A. Barassi , B. Venerando , and N. Papini , “Dexamethasone‐Induced Skeletal Muscle Atrophy Increases O‐GlcNAcylation in C2C12 Cells,” Journal of Cellular Biochemistry 117, no. 8 (2016): 1833–1842. 26728070 10.1002/jcb.25483

[24] S. A. Yuzwa , M. S. Macauley , J. E. Heinonen , et al., “A Potent Mechanism‐Inspired O‐GlcNAcase Inhibitor That Blocks Phosphorylation of Tau *In Vivo* ,” Nature Chemical Biology 4, no. 8 (2008): 483–490. 18587388 10.1038/nchembio.96

[25] K. Kamemura , B. K. Hayes , F. I. Comer , and G. W. Hart , “Dynamic Interplay Between O‐Glycosylation and O‐Phosphorylation of Nucleocytoplasmic Proteins: Alternative Glycosylation/Phosphorylation of THR‐58, a Known Mutational Hot Spot of c‐Myc in Lymphomas, Is Regulated by Mitogens,” Journal of Biological Chemistry 277, no. 21 (2002): 19229–19235. 11904304 10.1074/jbc.M201729200

[26] M. Ogawa , H. Mizofuchi , Y. Kobayashi , et al., “Terminal Differentiation Program of Skeletal Myogenesis Is Negatively Regulated by O‐GlcNAc Glycosylation,” Biochimica et Biophysica Acta 1820, no. 1 (2012): 24–32. 22056510 10.1016/j.bbagen.2011.10.011

[27] M. Ogawa , Y. Sakakibara , and K. Kamemura , “Requirement of Decreased O‐GlcNAc Glycosylation of Mef2D for Its Recruitment to the Myogenin Promoter,” Biochemical and Biophysical Research Communications 433, no. 4 (2013): 558–562. 23523791 10.1016/j.bbrc.2013.03.033

[28] K. Sun , Y. Zhi , W. Ren , et al., “Crosstalk Between O‐GlcNAcylation and Ubiquitination: A Novel Strategy for Overcoming Cancer Therapeutic Resistance,” Experimental Hematology & Oncology 13, no. 1 (2024): 107. 39487556 10.1186/s40164-024-00569-5 PMC11529444

[29] F. Zhang , K. Su , X. Yang , D. B. Bowe , A. J. Paterson , and J. E. Kudlow , “O‐GlcNAc Modification Is an Endogenous Inhibitor of the Proteasome,” Cell 115 (2003): 715–725. 14675536 10.1016/s0092-8674(03)00974-7

[30] M. Rechsteiner and S. W. Rogers , “PEST Sequences and Regulation by Proteolysis,” Trends in Biochemical Sciences 21, no. 7 (1996): 267–271. 8755249

[31] C. Guinez , A. M. Mir , V. Dehennaut , et al., “Protein Ubiquitination Is Modulated by O‐GlcNAc Glycosylation,” FASEB Journal 22, no. 8 (2008): 2901–2911. 18434435 10.1096/fj.07-102509

[32] N. E. Zachara , N. O'Donnell , W. D. Cheung , J. J. Mercer , J. D. Marth , and G. W. Hart , “Dynamic O‐GlcNAc Modification of Nucleocytoplasmic Proteins in Response to Stress. A Survival Response of Mammalian Cells,” Journal of Biological Chemistry 279, no. 29 (2004): 30133–30142. 15138254 10.1074/jbc.M403773200

[33] Z. Kazemi , H. Chang , S. Haserodt , C. McKen , and N. E. Zachara , “O‐Linked β‐*N*‐Acetylglucosamine (O‐GlcNAc) Regulates Stress‐Induced Heat Shock Protein Expression in a GSK‐_3_β‐Dependent Manner,” Journal of Biological Chemistry 285, no. 50 (2010): 39096–39107. 20926391 10.1074/jbc.M110.131102 PMC2998145

[34] C. H. Lin , C. C. Liao , M. Y. Chen , and T. Y. Chou , “Feedback Regulation of O‐GlcNAc Transferase Through Translation Control to Maintain Intracellular O‐GlcNAc Homeostasis,” International Journal of Molecular Sciences 22, no. 7 (2021): 3463. 33801653 10.3390/ijms22073463 PMC8037101

[35] Z. Cheng , N. Shang , X. Wang , et al., “Discovery of 4‐(Arylethynyl)Piperidine Derivatives as Potent Nonsaccharide O‐GlcNAcase Inhibitors for the Treatment of Alzheimer's Disease,” Journal of Medicinal Chemistry 67, no. 16 (2024): 14292–14312. 39109492 10.1021/acs.jmedchem.4c01132

[36] H. G. Selnick , J. F. Hess , C. Tang , et al., “Discovery of MK‐8719, a Potent O‐GlcNAcase Inhibitor as a Potential Treatment for Tauopathies,” Journal of Medicinal Chemistry 62, no. 22 (2019): 10062–10097. 31487175 10.1021/acs.jmedchem.9b01090

[37] B. Permanne , A. Sand , S. Ousson , et al., “O‐GlcNAcase Inhibitor ASN90 Is a Multimodal Drug Candidate for Tau and α‐Synuclein Proteinopathies,” ACS Chemical Neuroscience 13, no. 8 (2022): 1296–1314. 35357812 10.1021/acschemneuro.2c00057 PMC9026285

[38] C. Cieniewski‐Bernard , V. Montel , S. Berthoin , and B. Bastide , “Increasing O‐GlcNAcylation Level on Organ Culture of Soleus Modulates the Calcium Activation Parameters of Muscle Fibers,” PLoS ONE 7 (2012): e48218. 23110217 10.1371/journal.pone.0048218 PMC3480486

[39] J. Hedou , C. Cieniewski‐Bernard , Y. Leroy , J. C. Michalski , Y. Mounier , and B. Bestide , “O‐Linked N‐Acetylglucosaminylation Is Invlved in the Ca^2+^ Activation Properties of Rat Skeletal Muscle,” Journal of Biological Chemistry 282 (2007): 10360–10369. 17289664 10.1074/jbc.M606787200

[40] S. von Haehling , A. J. S. Coats , and S. D. Anker , “Ethical Guidelines for Publishing in the *Journal of Cachexia, Sarcopenia and Muscle*: Update 2023,” Journal of Cachexia, Sarcopenia and Muscle 14, no. 6 (2023): 2981–2983. 38148513 10.1002/jcsm.13420 PMC10751405

