# Supplementary material for: O‐GlcNAcase Inhibitor Improves Denervation‐Induced Muscle Atrophy in Mice
Source: J Cachexia Sarcopenia Muscle. 2025 Sep 12;16(5):e70066. doi: 10.1002/jcsm.70066 (PMC12426611; doi:10.1002/jcsm.70066)
Supplement: Supplementary file 1 — Table S1: Primary and secondary antibodies used in this study. [file JCSM-16-e70066-s002.pdf]

Supplemental methods

C2C12 cell culture

Cell culture studies were performed using mouse C2C12 myoblast cell lines purchased from the American Type Culture Collection (Manassas, VA) as previously described [1, 2]. The cells were cultured in growth medium composed of Dulbecco's Modified Eagle's Medium (Sigma-Aldrich, Louis, MO) containing 10% fetal bovine serum (Thermo Fisher Scientific, Waltham, MA) and 1% penicillin/streptomycin (Thermo Fisher Scientific) at 37°C with 5% CO_2 in air. After reaching full confluency, myoblasts were differentiated into myotubes using differentiation medium composed of Dulbecco's Modified Eagle's Medium containing 2% horse serum (Thermo Fisher Scientific) and 1% penicillin/streptomycin. The differentiation medium was replaced every 2 days. After 4 to 5 days of differentiation induction, myotubes formation was confirmed by light microscopy with morphological alignment, elongation, and fusion, as previously described [1].

Immediately after myotubes formation was confirmed, C2C12 myotubes were treated with the O-GlcNAcase (OGA) inhibitor thiamet G (10^{-8}, 10^{-7}, or 10^{-6} mol/l, 24 hours, Sigma-Aldrich) or MK-8719 (10^{-8}, 10^{-7}, or 10^{-6} mol/l, 24 hours, Selleck), or the glutamine-fructose-6-phosphate amido-transferase (GFAT) inhibitor 6-diazo-5-oxo-L-norleucine crystalline (DON, 10^{-5}, 10^{-4}, or 10^{-3} mol/l, 24 hours, Sigma-Aldrich), in Dulbecco's Modified Eagle's Medium without amino acids (Fujifilm Wako Pure Chemical Corporation, Osaka). Phosphate-buffered saline (PBS) was used as a vehicle, at a final volume concentration of 0.1%.

Transfection of small interfering RNA and adenoviruses

C2C12 myotubes were transfected with 1 nmol/l of small interfering RNA (siRNA) against mouse OGA (si-OGA; sense, 5-GCAGUAGGCAAGUUGCACAtt-3`, antisense, 5-UGUGCAACUUGCCUACUGCtg-3`) or scrambled siRNA (si-Scramble) using Lipofectamine RNAiMAX (Thermo Fisher Scientific) and cultured in Dulbecco's Modified Eagle's Medium without amino acids (Fujifilm Wako Pure Chemical Corporation) for 24 hours [1].

The overexpression of wild-type Akt and O-GlcNacylation-resistant Akt (T479A and T430A) was achieved by infection of adenoviruses designed and generated from VectorBuilder Inc. (Chicago, IL). C2C12 myotubes were transiently infected with adenoviruses for 48 hours in differentiation medium at 2 days of differentiation, as previously described with minor modification [3-5]. At 4 days of differentiation, the myotubes were further transfected with si-OGA and cultured in Dulbecco's Modified Eagle's Medium without amino acids (Fujifilm Wako Pure Chemical Corporation) for 24 hours.

Measurements of myotube diameter

Measurement of myotube diameters was performed as previously described [6]. Briefly, images of myotubes were obtained using an optical microscope (BZ-X800, Keyence, Osaka), and the diameters of individual myotubes were determined using BZ-X800 Analyzer software (Keyence). Average diameters of at least 100 myotubes were determined for each condition at three points separated by 50 μm along the myotube.

Animal experiments

Male C57BL/6J mice were purchased from CLEA Japan Inc. (Tokyo). Mice were used for experiments at 10 to 13 weeks of age (weight 25–27 g). Mice were maintained on a 12-hour light/dark cycle, with free access to standard chow and water. All mice were randomly assigned to different groups. Data acquisition and analysis were performed by investigators who were blinded to the group assignment. All procedures involving animals and animal care protocols were approved by the Committee on Ethics of Animal Experiments of Kyushu University Graduate School of Medicine and Pharmaceutical Sciences (study approval no.: A24-292-0) and were performed in accordance with the Guidelines for Animal Experiments of Kyushu University and the Guidelines for the Care and Use of Laboratory Animals published by the US National Institutes of Health (revised in 2011).

Sciatic denervation-induced muscle atrophy mouse model

As it is known that there are clear sex differences in the various characteristics of mice, including skeletal muscle weight [7], only male mice were used in the experiments. Many animal models are used in the study of skeletal muscle atrophy, including denervation, hindlimb unloading, and aging [8, 9]. The denervation model is widely used in research on skeletal muscle atrophy [10], especially as a model of atrophy caused by disuse. The denervation operation was performed by cutting the sciatic nerve on the right leg, and a sham operation was performed on the left leg of male C57BL/6J mice (n = 20) under anesthesia with 3% to 5% isoflurane (Viatris Inc., Canonsburg, PA) as described previously [11]. Mice were sacrificed by blood release under deep anesthesia with 5% isoflurane 1, 3, 5, and 7 days after the operation (n = 5 each), and

gastrocnemius muscles were excised, weighed, and used for histological and immunoblot analyses.

Mice (n = 26) that underwent the sham and denervation operation were randomly divided into 2 groups 3 days after the operation, and thiamet G (1 mg/kg body weight/day, Sigma-Aldrich) or vehicle (PBS) was continuously infused with an osmotic minipump (Alzet, Cupertino, CA). Fourteen mice were sacrificed 7 days and 12 mice were sacrificed 14 days after the operation, and gastrocnemius and soleus muscles were excised and analyzed in Sham + Vehicle, Sham + Thiamet G, Denervation + Vehicle, and Denervation + Thiamet G groups (n = 7 each for 7 days experiments, and n = 6 each for 14 days experiments). Furthermore, 20 male C57BL/6J mice underwent only the denervation operation. Three days after the operation, these mice were randomly divided into 4 groups (n = 5 each), and each group received continuous infusion of either 0.01, 0.1, or 1 mg/kg/day of thiamet G or vehicle for 4 days, and then they were sacrificed for analyses.

Adeno-associated virus serotype 1 (AAV1)-mediated suppression of OGA in the denervation-induced muscle atrophy model

To silence OGA expression, a short hairpin RNA (shRNA) sequence targeting OGA (sh-OGA; 5’- ATAGTGTCTGCAGGATTATTA-3’), and a scramble shRNA sequence (sh-Scramble; 5’- CCTAAGGTTAAGTCGCCCTCG-3’) were used. The U6 promoter was used to drive the expression of sh-OGA and sh-Scramble. These components were integrated into the AAV1 vectors under the control of the cytomegalovirus promoter. The AAV1 vectors were designed and generated by VectorBuilder. AAV1 vectors (1.0 $\times 10^{11}$ viral genomes in PBS) were injected intramuscularly into the gastrocnemius

muscle on both sides of male C57BL/6J mice, 7 days (n = 14) before denervation and sham operation [12]. Seven days after the operation (two weeks after viral injections), the two groups of mice (AAV1-sh-Scramble and AAV1-sh-OGA) were sacrificed as described above (n = 7).

Fasting-induced muscle atrophy model

Fasting-induced muscle atrophy was induced by the following protocol [13]. To carry out the experiments in similar conditions for fed and fasted mice, food was removed from the cages of all male C57BL/6J mice (n = 12) for 5 hours. After this period, food was given to all mice in the fed groups. Fasted mice were given food for 2 hours, and then the food was again removed for 48 hours until the experiments were performed. Animals were randomly divided into the fed and fasted groups (n = 6 each).

Male C57BL/6J mice (n = 14) were randomly divided into 2 groups after fasting for 24 hours, and thiamet G (10 mg/kg body weight) or vehicle (PBS) was administered intraperitoneally. The mice were then subjected to the grip strength test, and sacrificed 48 hours after fasting, and gastrocnemius muscles were excised and analyzed in the Fasted + Vehicle and Fasted + Thiamet G groups (n = 7 each).

Age-associated muscle atrophy mouse model

Male C57BL/6J mice aged 110 to 120 weeks were used as a model of age-associated muscle atrophy (n = 5), 13-week-old male C57BL/6J mice were used as young mice (n = 5).

Histological analyses

Excised gastrocnemius muscles were fixed in 10% formalin, embedded in paraffin, and stained with Picrosirius Red for histological analysis [14, 15]. The images of gastrocnemius muscle tissue were obtained using an optical microscope (BZ-X800). Myocyte cross-sectional area was analyzed in 100 to 150 myofibers from each mouse using BZ-X800 Analyzer software (Keyence) [4].

Grip strength test

Forelimb grip strength was evaluated using a triangular pull bar attached to a grip-strength meter (Columbus Instruments, Columbus, OH, USA). Each mouse was subjected to 5 consecutive tests to obtain the peak value, as previously described [13].

Western blotting

Western blotting was performed as previously described, with some modifications [4. 13, 16]. Frozen gastrocnemius and soleus muscles were homogenized with Cell Lysis Buffer (Cell Signaling Technology, Danvers, MA) supplemented with protease inhibitor cocktail (Roche, Basel), 1 mmol/l phenylmethylsulfonyl fluoride (Cell Signaling Technology), 4 μmol/l thiamet G (Sigma-Aldrich), and Halt Phosphatase Inhibitor Cocktail (Themo Fisher Scientific). Lysates were centrifuged at 13,200 *g* for 10 min at 4 °C, and the supernatants were collected. Protein concentrations were measured using a total protein assay (Pierce BCA, Rockford, IL). The samples were separated by sodium dodecyl sulfate-polyacrylamide gel electrophoresis, and proteins were transferred to nitrocellulose membranes at 100 volts for 1 hour. Membranes were blocked in 5% non-fat dry milk diluted in tris-buffered saline with 0.1% Tween-20 (TBST) for 1 hour at room temperature, and then incubated overnight

at 4 °C with the primary antibodies listed in **Supplemental Table 1**. After washing three times in TBST buffer, the membranes were incubated with secondary antibodies for 1 hour at room temperature. The membranes were washed again in TBST, and exposed proteins were visualized by the enhanced chemiluminescence method and signal intensities of the bands were quantified by Fusion Capt software (Vilber Lourmat, Marne-la-Vallée). Equal loading of protein samples was verified by staining with Coomassie Brilliant Blue (Nacalai Tesque, Kyoto). Quantification of protein levels was performed using the Image-J program (National Institutes of Health, Bethesda, MD).

Immunoprecipitation

Frozen gastrocnemius muscles were homogenized with Cell Lysis Buffer supplemented with protease inhibitor cocktail, 1 mmol/l phenylmethylsulfonyl fluoride, 40 μmol/l O-(2-acetamido-2-deoxy-D-glucopyranosylidenamino)N-phenylcarbamate (Sigma-Aldrich), and Halt Phosphatase Inhibitor Cocktail [17]. Lysates were centrifuged at 13,200 *g* for 10 min at 4 °C, and the supernatants were collected. Lysates were diluted to a protein concentration of 1 mg/ml with Cell Lysis Buffer, and incubated with an anti-O-GlcNAc antibody (RL-2) (NB300-524, Novus) or control IgG1 (Cell Signaling Technology, 5415) for 2 hours at 4 °C. Protein G beads were added to the lysates and incubated for 1 hour at 4 °C, as described previously [4]. Lysates were then centrifuged at 13,200 *g*, and pellets were washed with PBS and centrifuged again. This process was repeated 5 times. Pellets were then eluted by boiling for 5 minutes in Laemmli buffer (BioRad, Hercules, CA), and subjected to Western blotting to evaluate O-GlcNAcylation and Akt.

Quantitative real-time polymerase chain reaction (PCR)

Total RNA extraction and quantitative PCR were performed as described previously, with some modifications [4]. Briefly, total RNA was extracted using RNeasy Mini Kit (Qiagen, Hilden), the RNA was converted to cDNA using ReverTra Ace qPCR RT Kit (TOYOBO, Osaka), and the reactions were run in Applied Biosystems QuantStudio3 (Thermo Fisher Scientific, Waltham, MA) using THUNDERBIRD SYBR qPCR Mix (TOYOBO). The forward (F) and reverse (R) primer sequences were as follows:

ActB, F 5'- CACTGTCGAGTCGCGTCC -3', R 5'- TCATCCATGGCGAACTGGTG -3', and

OGA, F 5'-AGCAGCCAAGTTTGAAGAGA-3', R 5'-CCCTAACCACTGCACAAAAG -3'.

Statistical analyses

Data are expressed as the mean ± SD. No statistical method was used to predetermine the sample size. The number of animals used in each experiment was determined based on sample sizes commonly used in the field. The normality of the data was evaluated using the Shapiro-Wilk test. For two-group comparisons, the statistical evaluation of normally distributed variables was performed using the unpaired Student *t*-test, and the analysis of non-distributed variables was performed using Mann-Whitney *U* test. For three-group or four-group comparisons, statistical evaluation of normally distributed variables was performed using 1-way ANOVA followed by the Tukey test or Dunnett test as appropriate. If the data was non-distributed, analysis was performed using the Kruskal-Wallis test followed by Dunn's multiple comparisons test. For

comparisons of four groups with two factors (animal model; denervation × treatment; thiamet G or AAV1-sh-OGA), statistical analyses were performed using 2-way ANOVA, and when there was an interaction effect between two factors, the Tukey test was subsequently performed. Analyses were performed using GraphPad Prism 10 software (GraphPad, San Diego, CA). A *p*-value of less than 0.05 was considered to indicate a statistically significant difference between two groups.

Supplemental Table 1. Primary and secondary antibodies used in this study

Description	Dilution	Source	Identifier #
**Primary antibody**			
Anti-O-GlcNAc mouse	1:5000	Cell Signaling Technology	9875
Anti-O-GlcNAc transferase Rabbit	1:5000	Cell Signaling Technology	24083
Anti-OGA Rabbit	1:5000	Novus	NBP1-81244
Anti-GFAT1 Rabbit	1:1000	Abcam	ab125069
Anti-GFAT2 Rabbit	1:5000	Abcam	ab190966
Anti-Akt Rabbit	1:5000	Cell Signaling Technology	4691
Anti-phosphorylated Akt (S473) Rabbit	1:5000	Cell Signaling Technology	4060
Anti-phosphorylated Akt (T308) Rabbit	1:1000	Cell Signaling Technology	13038
Anti-mammalian target of rapamycin Rabbit	1:5000	Cell Signaling Technology	2983
Anti-phosphorylated mechanistic target of rapamycin (S2448) Rabbit	1:5000	Cell Signaling Technology	5536
Anti-p70S6 kinase Rabbit	1:5000	Cell Signaling Technology	2708
Anti-phosphorylated p70S6 kinase Rabbit	1:1000	Cell Signaling Technology	9234
Anti-forkhead box O3A Rabbit	1:1000	Cell Signaling Technology	2497
Anti-phosphorylated forkhead box O3A (Thr32) Rabbit	1:1000	Cell Signaling Technology	9464
Anti-muscle atrophy F-box Rabbit	1:5000	Abcam	ab16382
Anti-muscle RING Finger-1 Mouse	1:1000	Santacruz	sc398608
Anti-ubiquitin Mouse	1:5000	Santacruz	sc166553
Anti-hexokinase II Rabbit	1:5000	Abcam	ab209847
Anti-pyruvate kinase M Rabbit	1:5000	Abcam	ab137791
Anti-phosphorylated insulin receptor substrate 1 (Ser636/639) Rabbit	1:5000	Cell Signaling Technology	2388
Anti-insulin receptor substrate 1 Rabbit	1:5000	Cell Signaling Technology	3407
Anti-forkhead box O1 Rabbit	1:5000	Cell Signaling Technology	2880
Anti-phosphorylated forkhead box O1 (Ser256) Rabbit	1:5000	Cell Signaling Technology	9461
Anti-PGC1α Mouse	1:5000	Abcam	191838

Anti-AMP-activated protein kinase α Rabbit	1:5000	Cell Signaling Technology	2532
Anti-phosphorylated AMP-activated protein kinase α (Thr172) Rabbit	1:1000	Cell Signaling Technology	2535
Anti-Sirt1 Rabbit	1:5000	Cell Signaling Technology	9475
Anti-p62 Rabbit	1:5000	Cell Signaling Technology	23214
Anti-microtubule-associated protein light chain A/B Rabbit	1:5000	Cell Signaling Technology	4108
**Secondary antibody**			
Anti-rabbit IgG conjugated with horseradish peroxidase	1:5000	Cell Signaling Technology	7074
Anti-mouse IgG conjugated with horseradish peroxidase	1:5000	Cell Signaling Technology	7076

Figure S1. Inhibition of OGA increases the diameter of C2C12 myotubes

Representative photographs (left) and summary data (right) of the mean diameters of C2C12 myotubes transfected with si-Scramble or si-OGA (n = 6 in each group). Data are shown as the mean ± SD. *p*-values were calculated by the unpaired Student *t*-test. OGA, O-GlcNAcase; si, small interfering

Figure S2. MK-8719 enhances the phosphorylation of Akt and decreases the expression of muscle-specific ubiquitin ligases in C2C12 myotubes.

Representative western blots (left) and summary data (right) of O-GlcNAc (A), p-Akt (Ser473), Akt, atrogin-1 and MuRF1 (B) levels in C2C12 myotubes treated with PBS or different doses of MK-8719 (10^{-8}, 10^{-7}, and 10^{-6} mol/l) (n = 6 in each group). p-Akt was normalized to total Akt, and the other results were normalized to non-specific bands of the CBB-stained gel. Data are shown as the mean ± SD. *p*-values were calculated by 1-way ANOVA followed by the Dunnett *post hoc* test. PBS, phosphate-buffered saline; O-GlcNAc, O-linked N-acetylglucosamine; CBB, Coomassie Brilliant Blue; p-Akt, phosphorylated Akt; Atrogin-1, muscle atrophy F-box; MuRF-1, muscle RING Finger-1; N.S., not significant.

Figure S3. DON attenuates the phosphorylation of Akt and increases the expression of muscle-specific ubiquitin ligases in C2C12 myotubes

Representative western blots (left) and summary data (right) of O-GlcNAc (A), p-Akt (Ser473), Akt, atrogin-1 and MuRF1 (B) levels in C2C12 myotubes treated with PBS or different doses of DON (10^{-5}, 10^{-4}, and 10^{-3} mol/l) (n = 6 in each group). p-Akt was normalized to total Akt, and the other results were normalized to non-specific bands of

the CBB-stained gel. Data are shown as the mean ± SD. *p*-values were calculated by 1-way ANOVA followed by the Dunnett *post hoc* test, or the Kruskal-Wallis test followed by Dunn's *post hoc* test. PBS, phosphate-buffered saline; DON, diazo-5-oxo-L-norleucine; O-GlcNAc, O-linked N-acetylglucosamine; CBB, Coomassie Brilliant Blue; p-Akt, phosphorylated Akt; Atrogin-1, muscle atrophy F-box; MuRF-1, muscle RING Finger-1; N.S., not significant.

Figure S4. Changes in protein synthesis signaling in muscles undergoing denervation-induced atrophy.

Representative western blots (left) and summary data (right) of p-mTOR (Ser2448), mTOR, p-p70S6K (Thr389), and p70S6K levels in the gastrocnemius muscle of mice 1, 3, 5, and 7 days after denervation or sham operation (n = 4 in each group). Results were normalized to non-specific bands of the CBB-stained gel. Data are shown as the mean ± SD. *p*-values were calculated by 1-way ANOVA followed by the Dunnett *post hoc* test. Den, denervation; p-mTOR: phosphorylated mammalian target of rapamycin; p-p70S6K, phosphorylated p70 ribosomal S6 kinase; CBB, Coomassie Brilliant Blue; N.S., not significant.

Figure S5. Gene expression of O-GlcNAcase is increased in denervated gastrocnemius muscle

Summary data of the gene expression of *OGA* in the gastrocnemius muscle of sham or denervation (n=5 in each group). Gene expression was normalized to *ActB* gene expression. Data are shown as the mean ± SD. *p*-values were calculated by the Mann-Whitney U test. OGA: O-GlcNAcase.

Figure S6. Glycolytic enzyme levels do not change in skeletal muscle undergoing denervation-induced atrophy

Representative western blots (left) and summary data (right) of hexokinase II and pyruvate kinase M levels in the gastrocnemius muscle of mice 1, 3, 5, and 7 days after sham or denervation operation (n = 4 in each group). Results were normalized to non-specific bands of the CBB-stained gel. Data are shown as the mean ± SD. *p*-values were calculated by 1-way ANOVA followed by the Dunnett *post hoc* test, or the Kruskal-Wallis test followed by Dunn's *post hoc* test. Den, denervation; CBB, Coomassie Brilliant Blue; N.S., not significant.

Figure S7. Thiamet G treatment improves denervation-induced skeletal muscle atrophy in a dose-dependent manner.

(A) Experimental protocol for thiamet G treatment at different doses in mice with denervation-induced skeletal muscle atrophy. Summary data of body weight (B), gastrocnemius weight/body weight (C), and cross-sectional area of myocytes in gastrocnemius tissue sections (D) of mice treated with vehicle or thiamet G at different doses (0.01, 0.1, and 1 mg/kg/day) (n = 5 in each group). Data are shown as the mean ± SD. *p*-values were calculated by 1-way ANOVA followed by the Dunnett *post hoc* test, or Kruskal-Wallis test followed by Dunn's *post hoc* test. N.S., not significant.

Figure S8. Thiamet G treatment improves denervation-induced atrophy of soleus

(A) Summary data of soleus weight/body weight in Sham + Vehicle, Sham + Thiamet G, Denervation (Den) + Vehicle, and Den + Thiamet G groups (n = 7 in each group).

(B) Representative western blots (left) and summary data (right) of O-GlcNAc and OGA levels in soleus muscle of Sham + Vehicle, Denervation (Den) + Vehicle, and Den + Thiamet G groups (n = 7 in each group). Results were normalized to non-specific bands of the CBB-stained gel. Data are shown as the mean ± SD. In panel A, *p*-values of the main effect for each factor and interaction effect between two factors were calculated by 2-way ANOVA with the factors of denervation and thiamet G, and if there was an interaction effect between 2 factors, the Tukey *post hoc* test was performed. In panel B, *p*-values were calculated by 1-way ANOVA followed by the Tukey *post hoc* test. Den, denervation; O-GlcNAc, O-linked N-acetylglucosamine; OGA, OGlcNAcase; CBB, Coomassie Brilliant Blue

Figure S9. Thiamet G treatment for 2 weeks improves denervation-induced skeletal muscle atrophy

(A) Experimental protocol for thiamet G treatment of skeletal muscle undergoing denervation-induced atrophy. (B) Summary data of gastrocnemius weight/body weight (C) in Sham + Vehicle, Sham + Thiamet G, Denervation (Den) + Vehicle, and Den + Thiamet G groups (n = 6 in each group). *p*-values of the main effect for each factor and interaction effect between two factors were calculated by 2-way ANOVA with the factors of denervation and thiamet G, and if there was an interaction effect between 2 factors, the Tukey *post hoc* test was performed. Den, denervation

Figure S10. Insulin receptor substrate 1 (IRS1) level is not affected by thiamet G treatment

Representative western blots (left) and summary data (right) of IRS1and p-IRS1 (Ser636/639) levels in the gastrocnemius muscle of Sham + Vehicle, Denervation (Den) + Vehicle, and Den + Thiamet G (n = 7 in each group). Results were normalized to non-specific bands of the CBB-stained gel. Data are shown as the mean ± SD. *p*-values were calculated by 1-way ANOVA followed by the Tukey *post hoc* test or Kruskal-Wallis test followed by Dunn's *post hoc* test. Den, denervation; p-IRS1, phosphorylated insulin receptor substrate 1; CBB, Coomassie Brilliant Blue; N.S., not significant.

Figure S11. Expression of proteins associated with FoxO1, mitochondrial biogenesis, and autophagy

Representative western blots (A) and summary data (B) of p-FoxO1, FoxO1, and FoxO1/p-FoxO1, (C) PGC-1α, p-AMPKα/AMPKα, and Sirt1, (D) p62, LC3-I, and LC3-II in the gastrocnemius muscle of Sham + Vehicle (n = 7), Denervation (Den) + vehicle (n = 7), and Den + Thiamet G (n = 7). The blots were normalized to the nonspecific bands of CBB-stained gel. Data are shown as the mean ± SD. *p*-values were calculated by 1-way ANOVA followed by the Tukey *post hoc* test. Den, denervation; p-FoxO1, phosphorylated forkhead box O1; PGC-1α, peroxisome proliferator-activated receptor γ coactivator-1 α; p-AMPKα, phosphorylated AMP-activated protein kinase α; Sirt1, sirtuin 1; LC3, light chain 3; CBB, Coomassie Brilliant Blue.

Figure S12. O-GlcNAcylation of Akt is reduced in fasting-induced muscle atrophy

Summary data of body weight (A) and gastrocnemius weight/tibial length (B) in fed and fasted mice (n = 6 in each group). (C) Representative high-magnification photomicrographs (left) and summary data (right) of gastrocnemius tissue sections

stained with Picrosirius Red of the two groups (n = 6 in each group). Representative western blots (left) and summary data (right) of p-Akt (Ser473) and Akt (D), and O-GlcNAc and OGA (E) levels of the two groups (n = 6 in each group). Results were normalized to non-specific bands of the CBB-stained gel. (F) Immunoprecipitation assays using gastrocnemius muscle lysates from the two groups (n = 6 in each group). After immunoprecipitation with control IgG or an O-GlcNAc antibody, immunoblotting for Akt and O-GlcNAc was performed. Representative western blots (left) and summary of the data (right) of O-GlcNAc-Akt are shown. Data are shown as the mean ± SD. *p*-values were calculated by the unpaired Student *t*-test. p-Akt, phosphorylated Akt; O-GlcNAc, O-linked N-acetylglucosamine; IP, immunoprecipitation; IB, immunoblotting; O-GlcNAc-Akt, O-GlcNAcylated Akt; N.S., not significant.

Figure S13. O-GlcNAcylation of Akt is reduced in age-associated muscle atrophy

Summary data of body weight (A) and gastrocnemius weight/tibial length (B) in young and aged mice (n = 5 in each group). (C) Representative high-magnification photomicrographs (left) and summary data (right) of the cross-sectional area of myocytes in gastrocnemius tissue sections stained with Picrosirius Red of the two groups (n = 6 in each group). Representative western blots (top) and summary data (bottom) of p-Akt (Ser473) and Akt (D) in the two groups (n = 6 in each group). Representative western blots (left) and summary data (right) of O-GlcNAc, OGT, and OGA (E) levels in the two groups (n = 6, 3, 3 in each group). Results were normalized to non-specific bands of the CBB-stained gel. (F) Immunoprecipitation assays using gastrocnemius lysates of in the two groups (n = 6 in each group). After immunoprecipitation with control IgG or an O-GlcNAcylation antibody,

immunoblotting for Akt and O-GlcNAc was performed. Representative western blots (left) and summary data (right) of O-GlcNAc-Akt are shown. Data are shown as the mean ± SD. *p*-values were calculated by the unpaired Student *t*-test. p-Akt, phosphorylated Akt; O-GlcNAc, O-linked N-acetylglucosamine; IP, immunoprecipitation; IB, immunoblotting; O-GlcNAc-Akt, O-GlcNAcylated Akt; N.S., not significant.

Figure S14. No interaction between phosphorylation at serine 473 and O-GlcNAcylation of Akt at threonine 430 in C2C12 myotubes

(A) Representative immunoblotting (left) and summary data (right) of p-Akt (Ser473 and Thr308) and Akt levels in C2C12 myotubes treated with an adenovirus expressing wild-type Akt (Ad-Akt wt) or mutant Akt (T430A) (Ad-Akt T430A), transfected with a small-interfering RNA against O-GlcNAcase (si-OGA) (n = 6 in each group). p-Akt was normalized to total Akt. Immunoblots in C2C12 myotubes transfected with si-Scramble were also shown. (B) Immunoprecipitation assays using lysates of C2C12 myotubes in two groups (n = 6 in each group). After immunoprecipitation with control IgG or an O-GlcNAc antibody, immunoblotting for Akt and O-GlcNAc was performed. Representative western blots (left) and summary data (right) of O-GlcNAc-Akt are shown. Data are shown as the mean ± SD. *p*-values were calculated by the unpaired Student *t*-test or Mann-Whitney U test. ad, adenovirus; wt, wild-type; p-Akt, phosphorylated Akt, OGA, O-GlcNAcase; CBB, Coomassie Brilliant Blue; siRNA, small interfering RNA; IP, immunoprecipitation; IB, immunoblotting; O-GlcNAc, O-linked N-acetylglucosamine; O-GlcNAc-Akt, O-GlcNAcylated Akt; N.S., not significant.

Supplemental references

[1] Fukushima A, Kinugawa S, Takada S, Matsushima S, Sobirin MA, Ono T, et al. (Pro)renin receptor in skeletal muscle is involved in the development of insulin resistance associated with postinfarct heart failure in mice. *Am J Physiol Endocrinol Metab*. 2014;**307**(6):E503-14.

[2] Matsumoto J, Takada S, Furihata T, Nambu H, Kakutani N, Maekawa S, et al. Brain-derived neurotrophic factor improves impaired fatty acid oxidation via the activation of adenosine monophosphate-activated protein kinase-α - proliferator-activated receptor-γ coactivator-1α signaling in skeletal muscle of mice with heart failure. *Circ Heart Fail*. 2021;**14**(1):e005890.

[3] Li F, Xu M, Miao J, Hu N, Wang Y, Wang L. Down-regulated Smyd1 participated in the inhibition of myoblast differentiation induced by cigarette smoke extract. *Toxicol Lett*. 2023;**383**:98-111.

[4] Ishikita A, Matsushima S, Ikeda S, Okabe K, Nishimura R, Tadokoro T, et al. GFAT2 mediates cardiac hypertrophy through HBP-O-GlcNAcylation-Akt pathway. *iScience*. 2021;**24**(12):103517.

[5] Wang Q, Moncman CL, Winkelmann DA. Mutations in the motor domain modulate myosin activity and myofibril organization. *J Cell Sci*. 2003;**116**(Pt 20):4227-38.

[6] Chang JS, Kong ID. Irisin prevents dexamethasone-induced atrophy in C2C12 myotubes. *Pflugers Arch*. 2020;**472**(4):495-502.

[7] O’Reilly J, Ono-Moore KD, Chintapalli SV, Rutkowsky JM, Tolentino T, Lloyd KKC, et al. Sex differences in skeletal muscle revealed through fiber type, capillarity, and transcriptomics profiling in mice. *Physiol Rep*. 2021;**9**(18):e15031.

[8] Xie WQ, He M, Yu DJ, Wu YX, Wang XH, Lv S, et al. Mouse models of sarcopenia: classification and evaluation. *J Cachexia Sarcopenia Muscle*. 2021;**12**:538-554.

[9] Palus S, Springer JI, Doehner W, von Haehling S, Anker M, Anker SD, et al. Models of sarcopenia: Short review. *Int J Cardiol*. 2017;**238**:19-21.

[10] Chiu HC, Chiu CY, Yang RS, Chan DC, Liu SH, Chiang CK. Preventing muscle wasting by osteoporosis drug alendronate in vitro and in myopathy models via sirtuin-3 down-regulation. *J Cachexia Sarcopenia Muscle*. 2018;**9**:585-602.

[11] Tokinoya K, Shirai T, Ota Y, Takemasa T, Takekoshi K. Denervation-induced muscle atrophy suppression in renalase-deficient mice via increased protein synthesis. *Physiol Rep*. 2020;**8**(15):e14475.

[12] Ozes B, Tong L, Myers M, Moss K, Ridgley A, Sahenk Z. AAV1.NT-3 gene therapy prevents age-related sarcopenia. *Aging*. 2023;**15**(5):1306-29.

[13] de Vasconcelos DAA, Giesbertz P, de Souza DR, Vitzel KF, Abreu P, Marzuca-Nassr GN, et al. Oral L-glutamine pretreatment attenuates skeletal muscle atrophy induced by 24-h fasting in mice. *J Nutr Biochem*. 2019;**70**:202-14.

[14] Yamanashi K, Kinugawa S, Fukushima A, Kakutani N, Takada S, Obata Y, et al. Branched-chain amino acid supplementation ameliorates angiotensin II-induced skeletal muscle atrophy. *Life Sci*. 2020;**250**:117593.

[15] Kakutani N, Takada S, Nambu H, Matsumoto J, Furihata T, Yokota T, et al. Angiotensin-converting-enzyme inhibitor prevents skeletal muscle fibrosis in myocardial infarction mice. *Skelet Muscle*. 2020;**10**(1):11.

[16] Nambu H, Takada S, Fukushima A, Matsumoto J, Kakutani N, Maekawa S, et al. Empagliflozin restores lowered exercise endurance capacity via the activation of

skeletal muscle fatty acid oxidation in a murine model of heart failure. *Eur J Pharmacol*. 2020;**866**:172810.

[17] Shi H, Munk A, Nielsen TS, Daughtry MR, Larsson L, Li S, et al. Skeletal muscle O-GlcNAc transferase is important for muscle energy homeostasis and whole-body insulin sensitivity. *Mol Metab*. 2018;**11**:160-77.
