# Supplementary figures and images for: O‐GlcNAcase Inhibitor Improves Denervation‐Induced Muscle Atrophy in Mice
Source: J Cachexia Sarcopenia Muscle. 2025 Sep 12;16(5):e70066. doi: 10.1002/jcsm.70066 (PMC12426611; doi:10.1002/jcsm.70066)

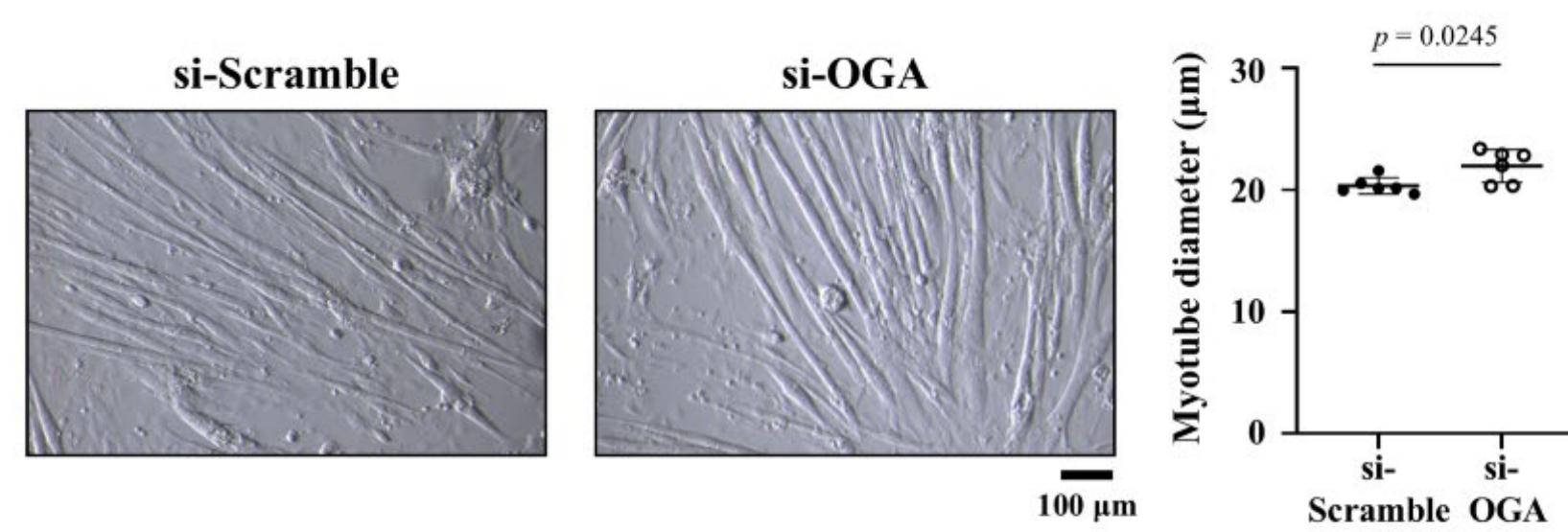

Figure S1

Supplement: Supplementary file 2 — Figure S1: Inhibition of OGA increases the diameter of C2C12 myotubes. Representative photographs (left) and summary data (right) of the mean diameters of C2C12 myotubes transfected with si‐Scramble or si‐OGA (n = 6 in each group). Data are shown as the mean ± SD. p values were calculated by the unpaired Student t‐test. OGA, O‐GlcNAcase; si, small interfering. [file JCSM-16-e70066-s007.pdf]

**A**

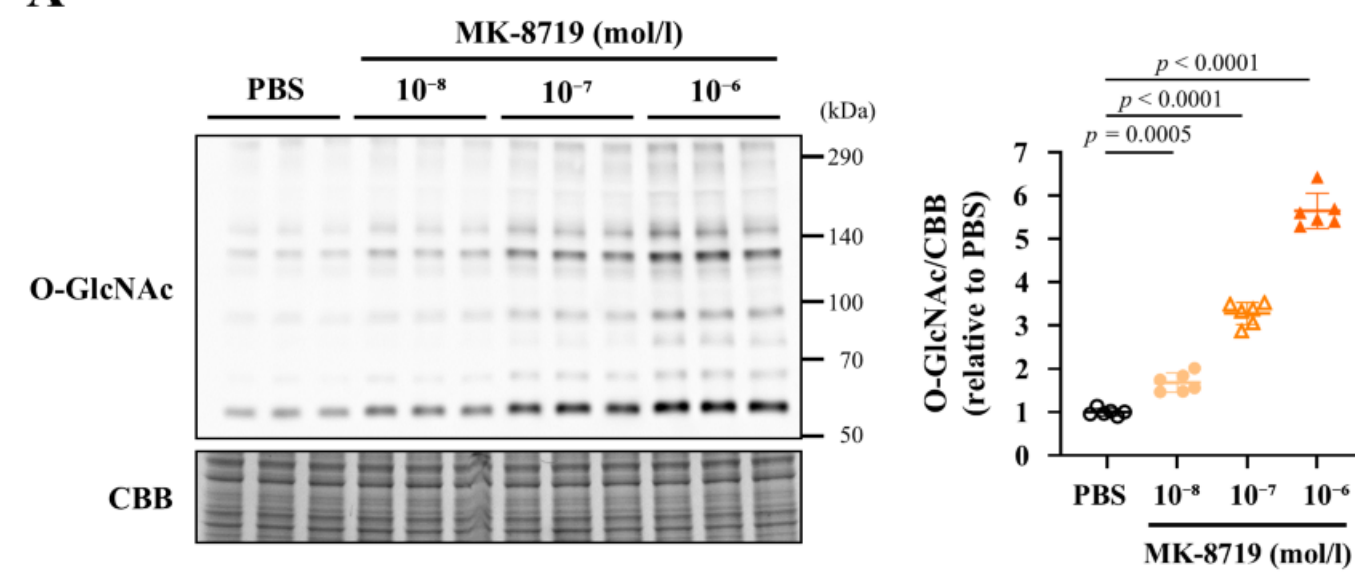

**B**

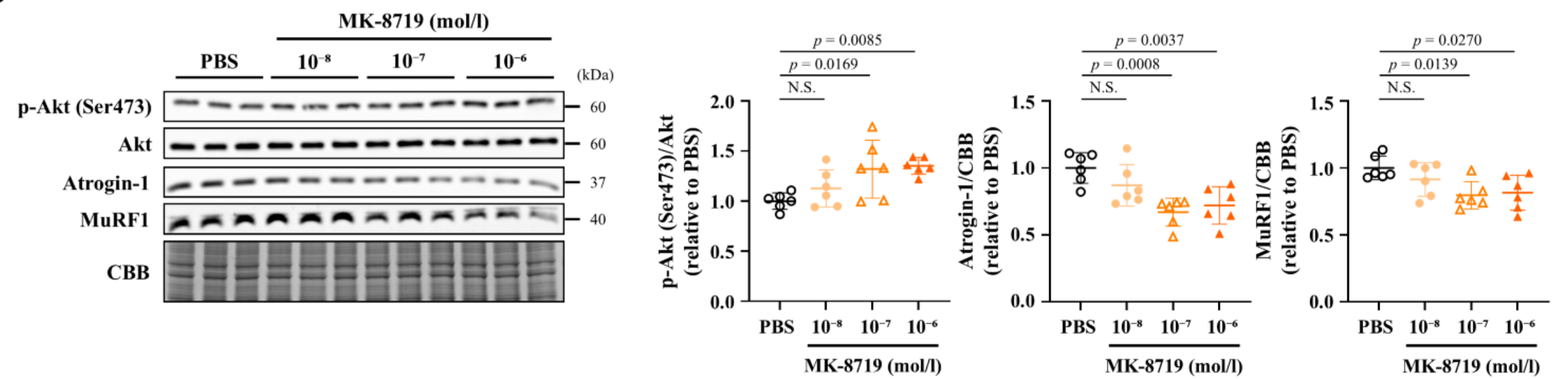

Figure S2

Supplement: Supplementary file 3 — Figure S2: MK‐8719 enhances the phosphorylation of Akt and decreases the expression of muscle‐specific ubiquitin ligases in C2C12 myotubes. Representative western blots (left) and summary data (right) of O‐GlcNAc (A), p‐Akt (Ser473), Akt, atrogin‐1 and MuRF1 (B) levels in C2C12 myotubes treated with PBS or different doses of MK‐8719 (10−8, 10−7 and 10−6 mol/L) (n = 6 in each group). p‐Akt was normalized to total Akt, and the other results were normalized to non‐specific bands of the CBB‐stained gel. Data are shown as the mean ± SD. p values were calculated by one‐way ANOVA, followed by the Dunnett post hoc test. Atrogin‐1, muscle atrophy F‐box; CBB, Coomassie Brilliant Blue; MuRF‐1, muscle RING Finger‐1; NS, not significant; O‐GlcNAc, O‐linked N‐acetylglucosamine; p‐Akt, phosphorylated Akt; PBS, phosphate‐buffered saline. [file JCSM-16-e70066-s013.pdf]

**A**

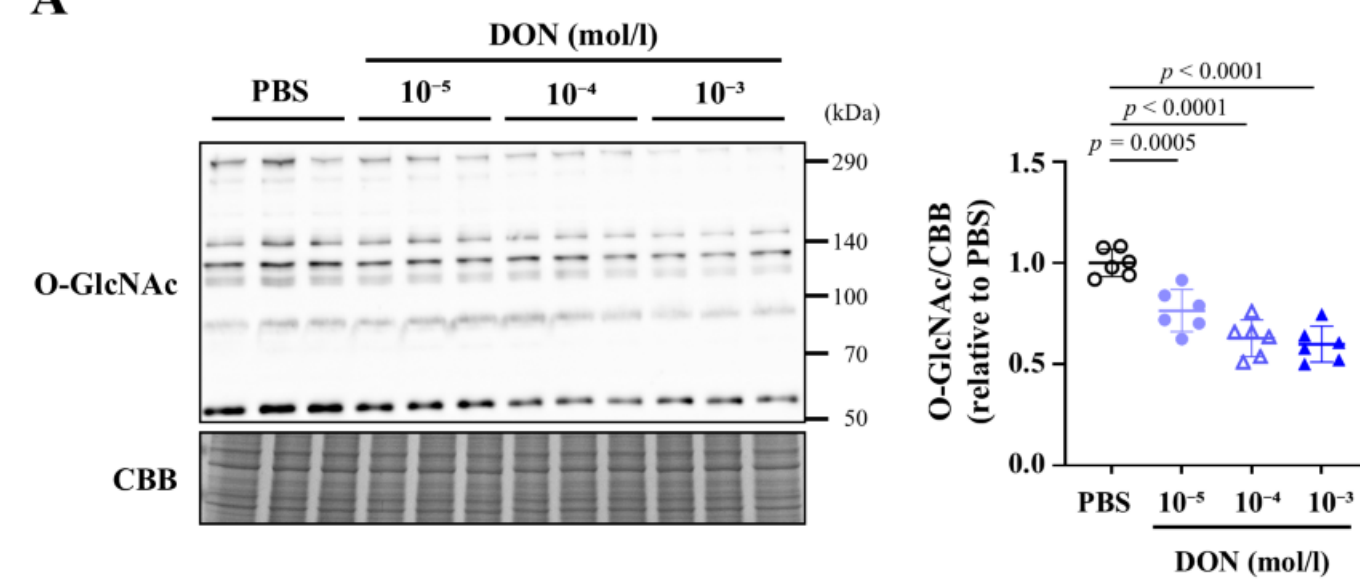

**B**

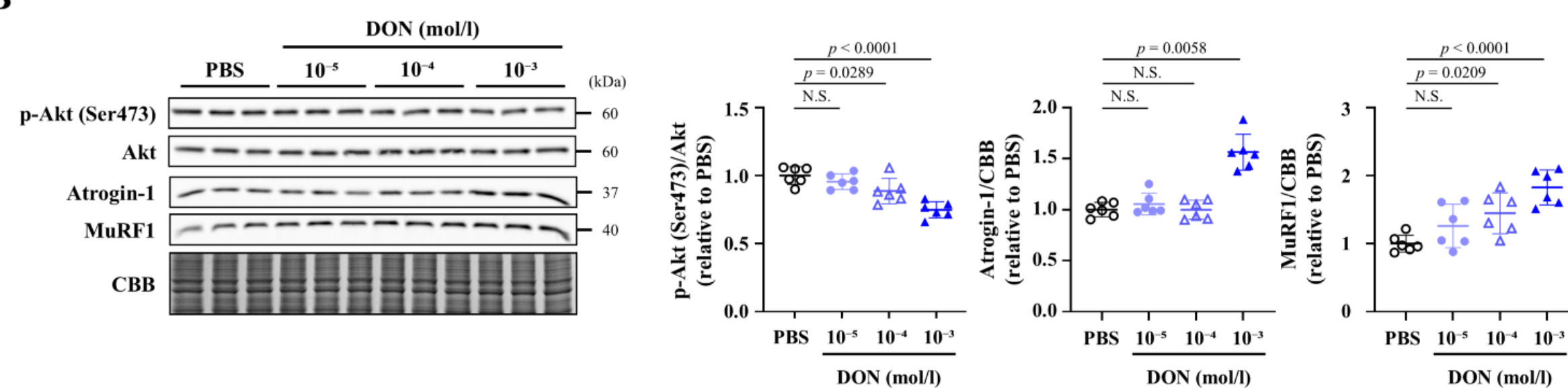

Figure S3

Supplement: Supplementary file 4 — Figure S3: DON attenuates the phosphorylation of Akt and increases the expression of muscle‐specific ubiquitin ligases in C2C12 myotubes. Representative western blots (left) and summary data (right) of O‐GlcNAc (A), p‐Akt (Ser473), Akt, atrogin‐1 and MuRF1 (B) levels in C2C12 myotubes treated with PBS or different doses of DON (10−5, 10−4 and 10−3 mol/L) (n = 6 in each group). p‐Akt was normalized to total Akt, and the other results were normalized to non‐specific bands of 13 the CBB‐stained gel. Data are shown as the mean ± SD. p values were calculated by one‐way ANOVA, followed by the Dunnett post hoc test or the Kruskal–Wallis test, followed by Dunn's post hoc test. Atrogin‐1, muscle atrophy F‐box; CBB, Coomassie Brilliant Blue; DON, diazo‐5‐oxo‐L‐norleucine; MuRF‐1, muscle RING Finger‐1; NS, not significant; O‐GlcNAc, O‐linked N‐acetylglucosamine; p‐Akt, phosphorylated Akt; PBS, phosphate‐buffered saline. [file JCSM-16-e70066-s008.pdf]

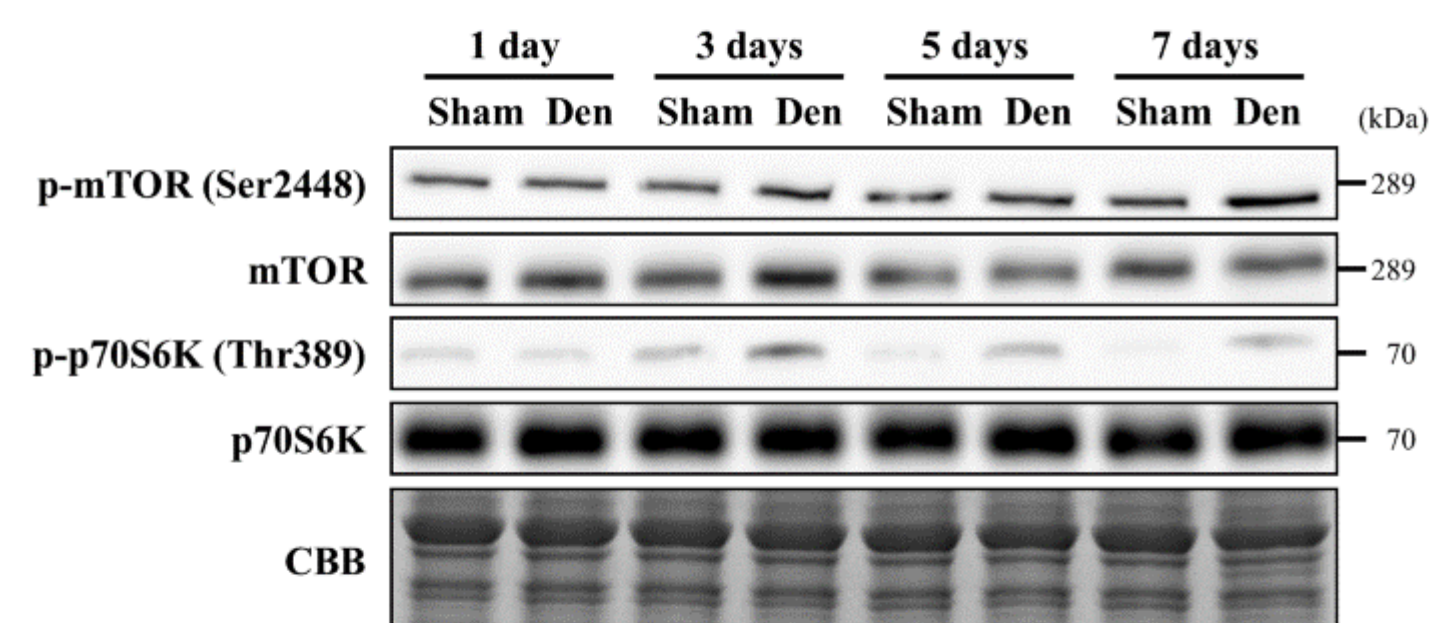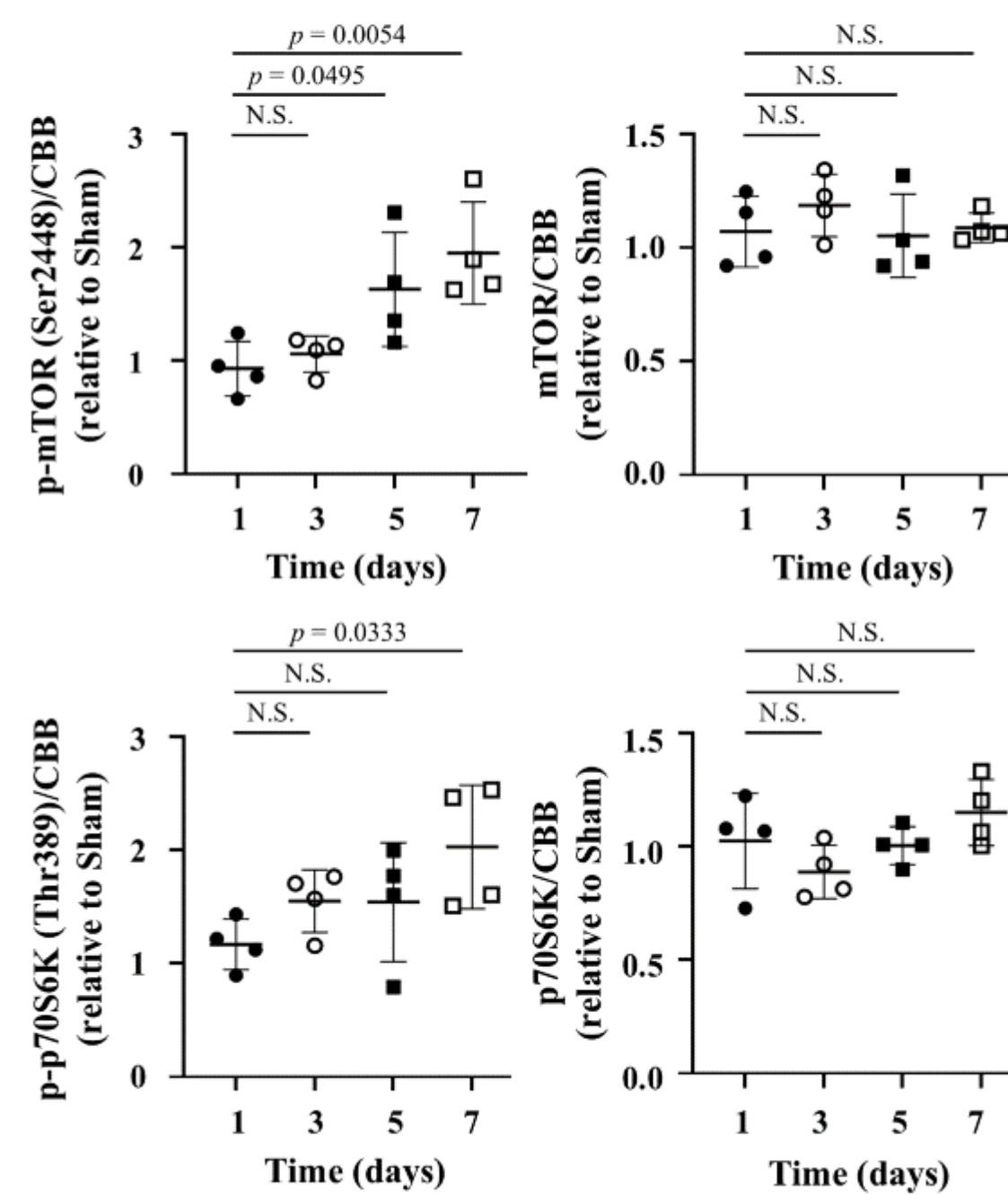

Figure S4

Supplement: Supplementary file 5 — Figure S4: Changes in protein synthesis signalling in muscles undergoing denervation‐induced atrophy. Representative western blots (left) and summary data (right) of p‐mTOR (Ser2448), mTOR, p‐p70S6K (Thr389) and p70S6K levels in the gastrocnemius muscle of mice 1, 3, 5 and 7 days after denervation or sham operation (n = 4 in each group). Results were normalized to non‐specific bands of the CBB‐stained gel. Data are shown as the mean ± SD. p values were calculated by one‐way ANOVA, followed by the Dunnett post hoc test. CBB, Coomassie Brilliant Blue; Den, denervation; NS, not significant; p‐p70S6K, phosphorylated p70 ribosomal S6 kinase; p‐mTOR, phosphorylated mammalian target of rapamycin. [file JCSM-16-e70066-s004.pdf]

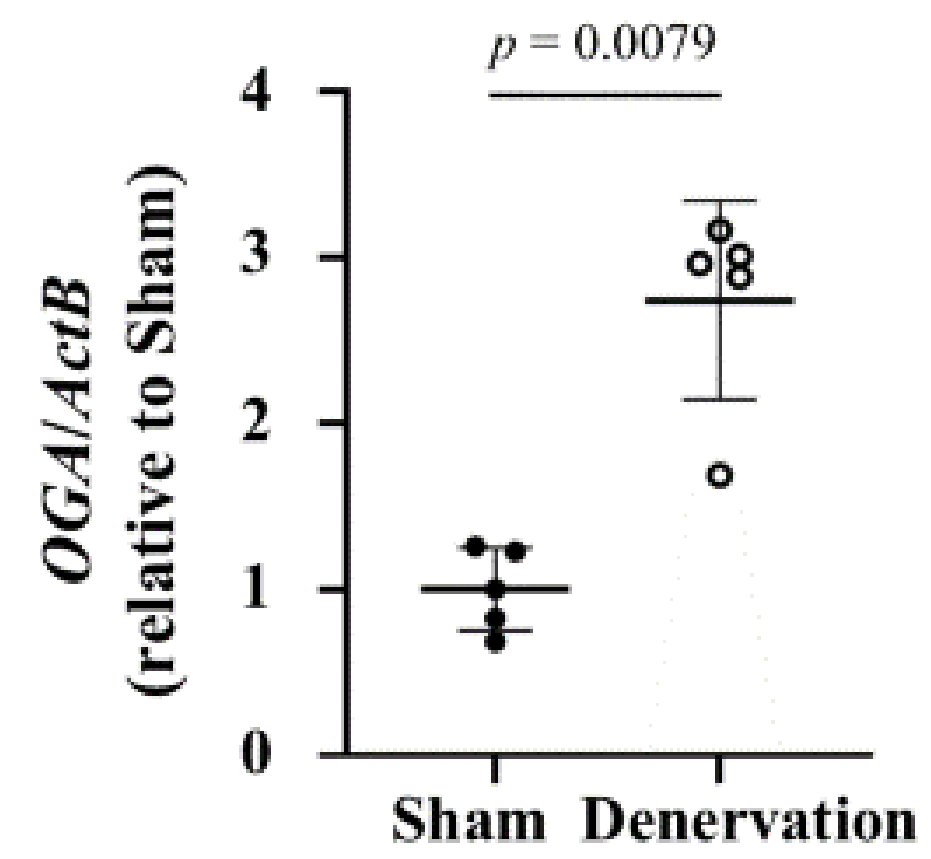

Figure S5

Supplement: Supplementary file 6 — Figure S5: Gene expression of O‐GlcNAcase is increased in denervated gastrocnemius muscle Summary data of the gene expression of OGA in the gastrocnemius muscle of sham or denervation (n = 5 in each group). Gene expression was normalized to ActB gene expression. Data are shown as the mean ± SD. p values were calculated by the Mann–Whitney U test. OGA, O‐GlcNAcase. [file JCSM-16-e70066-s010.pdf]

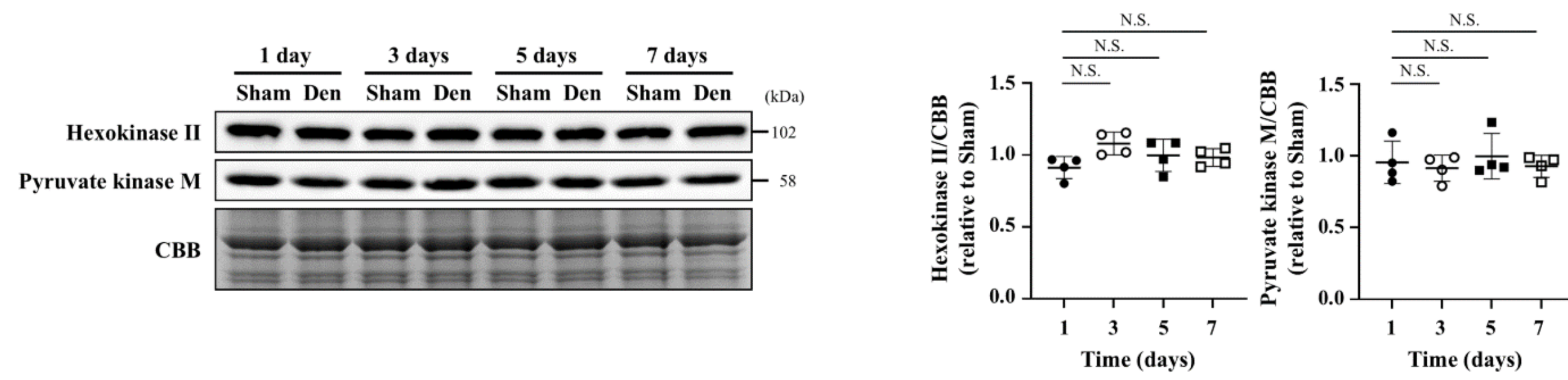

Figure S6

Supplement: Supplementary file 7 — Figure S6: Glycolytic enzyme levels do not change in skeletal muscle undergoing denervation‐induced atrophy. Representative western blots (left) and summary data (right) of hexokinase II and pyruvate kinase M levels in the gastrocnemius muscle of mice 1, 3, 5 and 7 days after sham or denervation operation (n = 4 in each group). Results were normalized to non‐specific bands of the CBB‐stained gel. Data are shown as the mean ± SD p‐values were calculated by one‐way ANOVA, followed by the Dunnett post hoc test, or the Kruskal–Wallis test followed by Dunn's post hoc test. CBB, Coomassie Brilliant Blue; Den, denervation; NS, not significant. [file JCSM-16-e70066-s006.pdf]

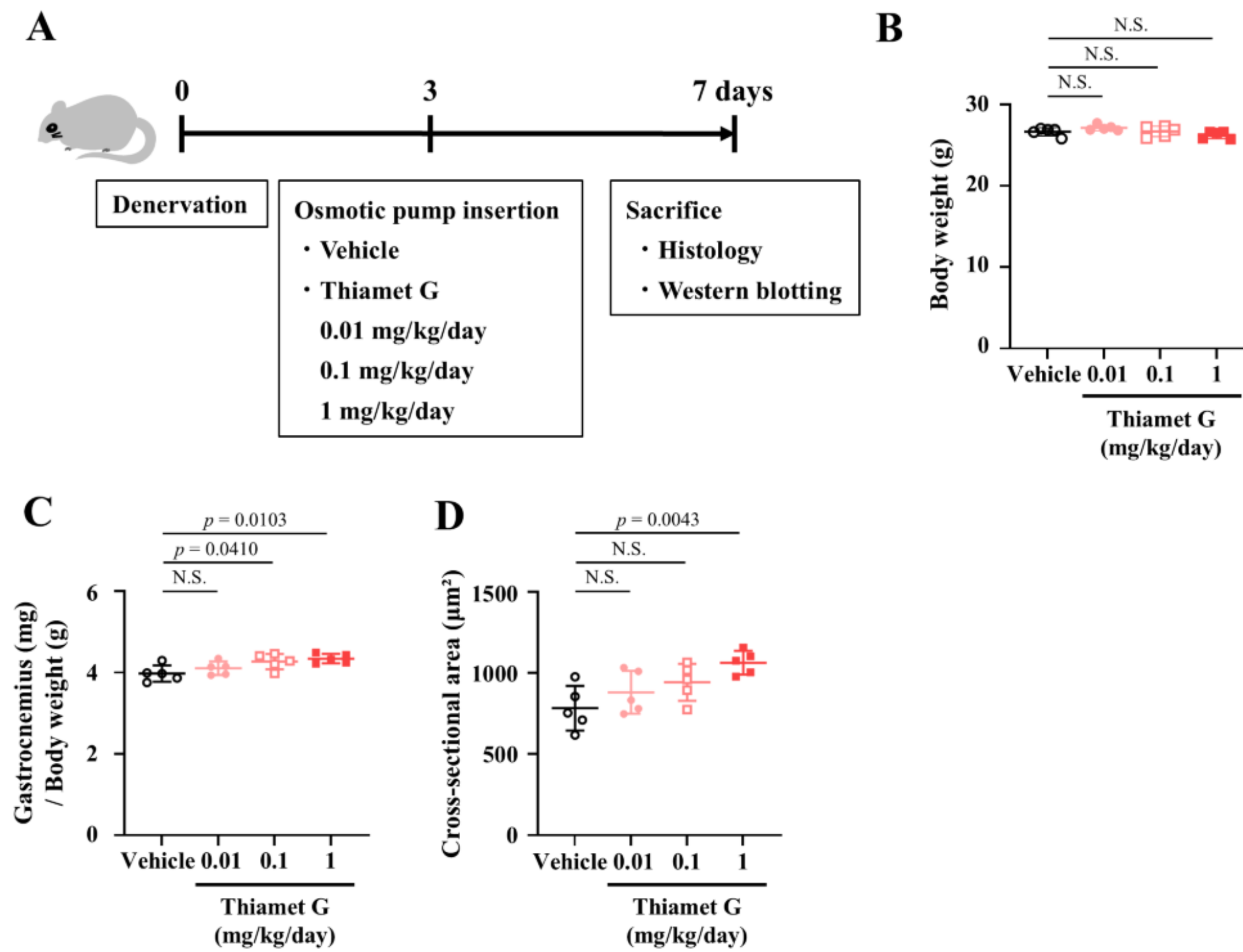

Figure S7

Supplement: Supplementary file 8 — Figure S7: Thiamet G treatment improves denervation‐induced skeletal muscle atrophy in a dose‐dependent manner. (A) Experimental protocol for thiamet G treatment at different doses in mice with denervation‐induced skeletal muscle atrophy. Summary data of body weight (B), gastrocnemius weight/body weight (C) and cross‐sectional area of myocytes in gastrocnemius tissue sections (D) of mice treated with vehicle or thiamet G at different doses (0.01, 0.1 and 1 mg/kg/day) (n = 5 in each group). Data are shown as the mean ± SD. p values were calculated by one‐way ANOVA followed by the Dunnett post hoc test, or Kruskal–Wallis test followed by Dunn's post hoc test. NS, not significant. [file JCSM-16-e70066-s015.pdf]

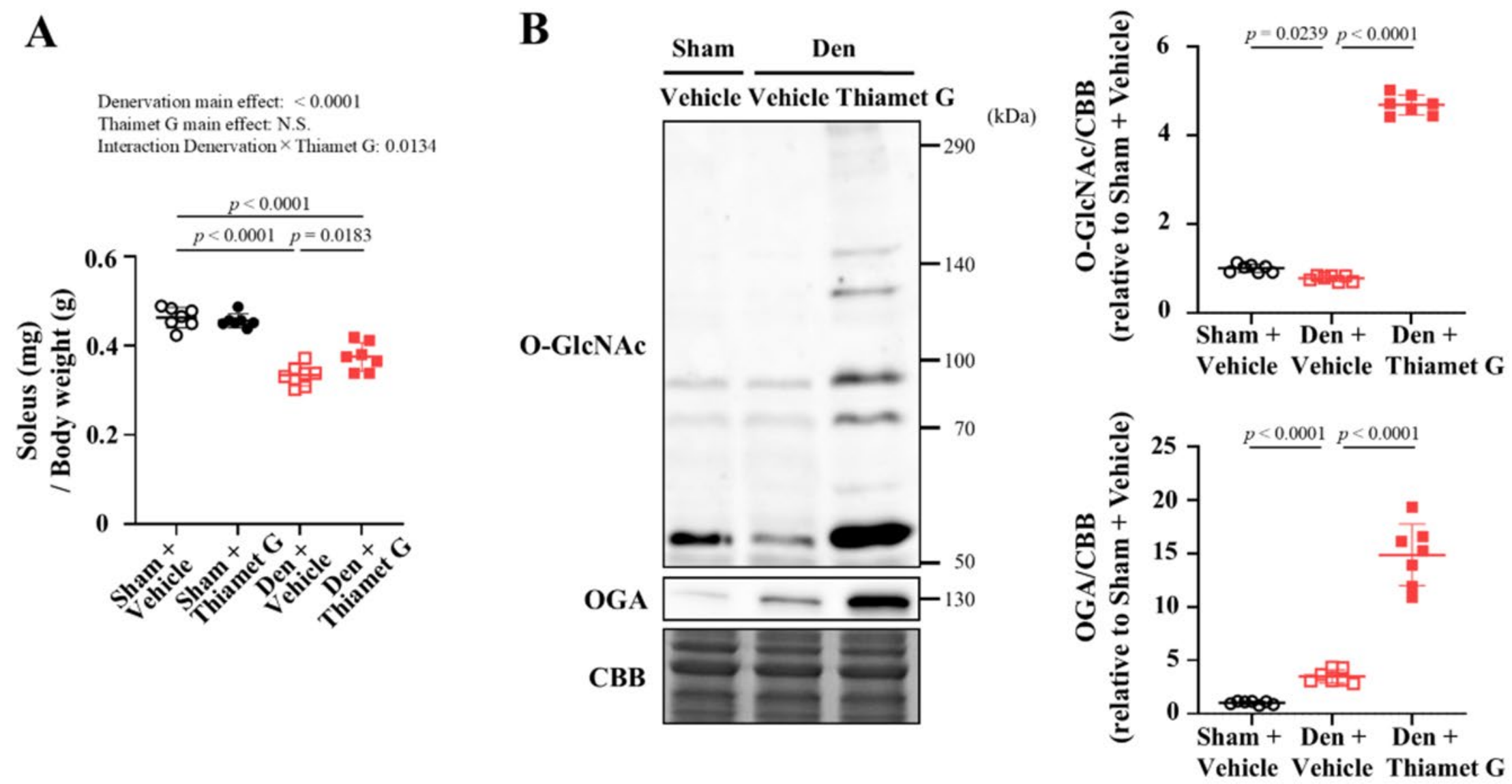

Figure S8

Supplement: Supplementary file 9 — Figure S8: Thiamet G treatment improves denervation‐induced atrophy of soleus. (A) Summary data of soleus weight/body weight in sham + vehicle, sham + thiamet G, denervation (Den) + vehicle and Den + thiamet G groups (n = 7 in each group). (B) Representative western blots (left) and summary data (right) of O‐GlcNAc and OGA levels in soleus muscle of sham + vehicle, denervation (Den) + vehicle and Den + thiamet G groups (n = 7 in each group). Results were normalized to non‐specific bands of the CBB‐stained gel. Data are shown as the mean ± SD. In Panel A, p values of the main effect for each factor and interaction effect between two factors were calculated by two‐way ANOVA with the factors of denervation and thiamet G, and if there was an interaction effect between two factors, the Tukey post hoc test was performed. In Panel B, p values were calculated by one‐way ANOVA followed by the Tukey post hoc test. CBB, Coomassie Brilliant Blue; Den, denervation; OGA, OGlcNAcase; O‐GlcNAc, O‐linked N‐acetylglucosamine. [file JCSM-16-e70066-s001.pdf]

**A**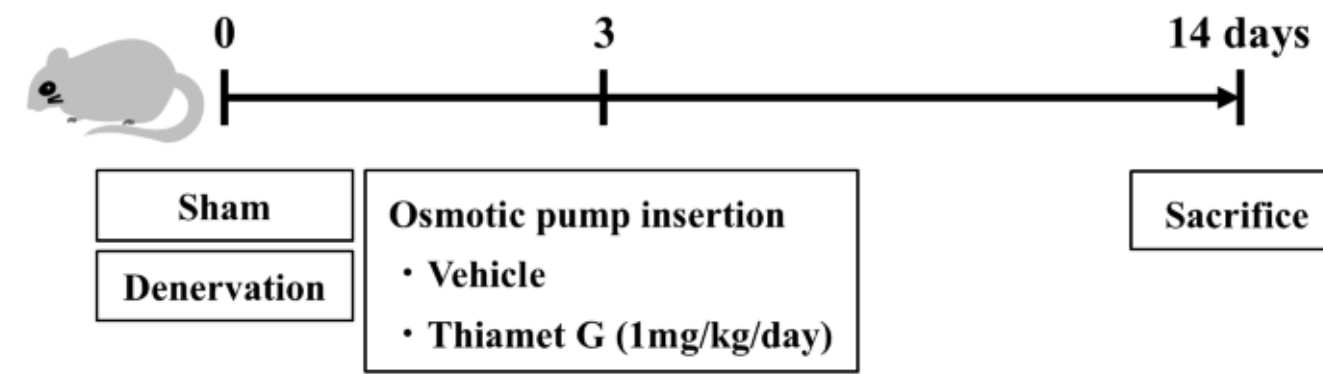**B**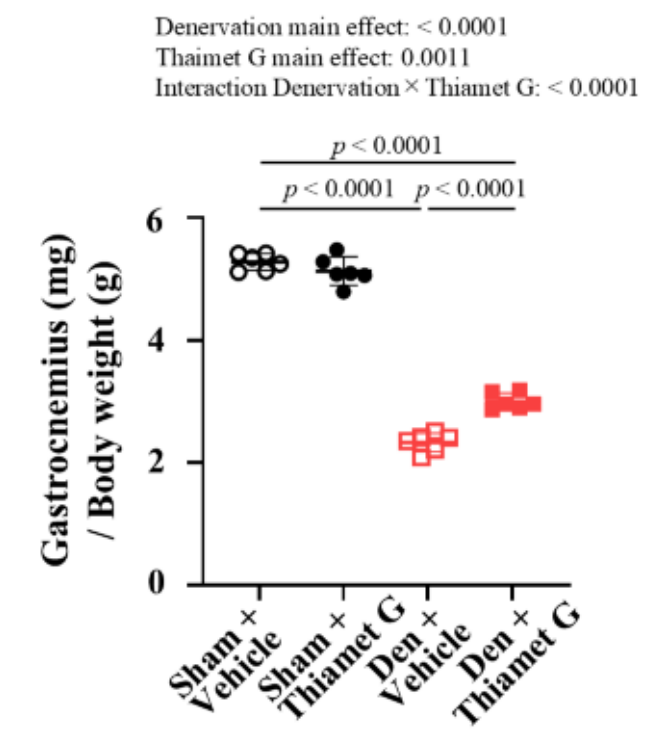

Figure S9

Supplement: Supplementary file 10 — Figure S9: Thiamet G treatment for 2 weeks improves denervation‐induced skeletal muscle atrophy. (A) Experimental protocol for thiamet G treatment of skeletal muscle undergoing denervation‐induced atrophy. (B) Summary data of gastrocnemius weight/body weight (C) in sham + vehicle, sham + thiamet G, denervation (Den) + vehicle and Den + thiamet G groups (n = 6 in each group). p values of the main effect for each factor and interaction effect between two factors were calculated by two‐way ANOVA with the factors of denervation and thiamet G, and if there was an interaction effect between two factors, the Tukey post hoc test was performed. Den, denervation. [file JCSM-16-e70066-s012.pdf]

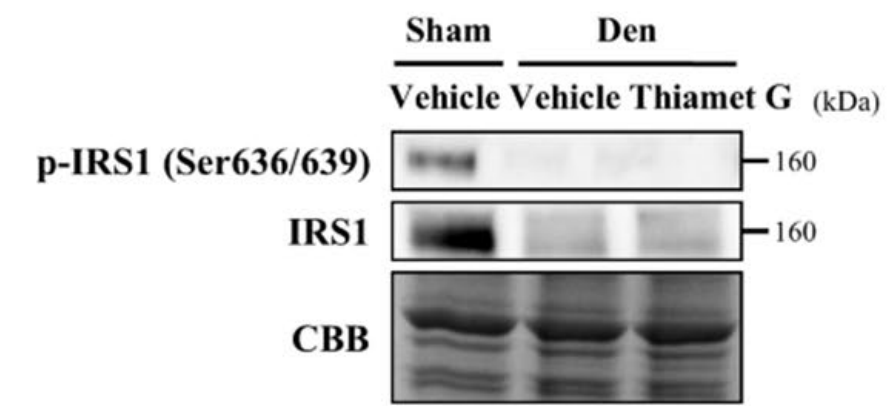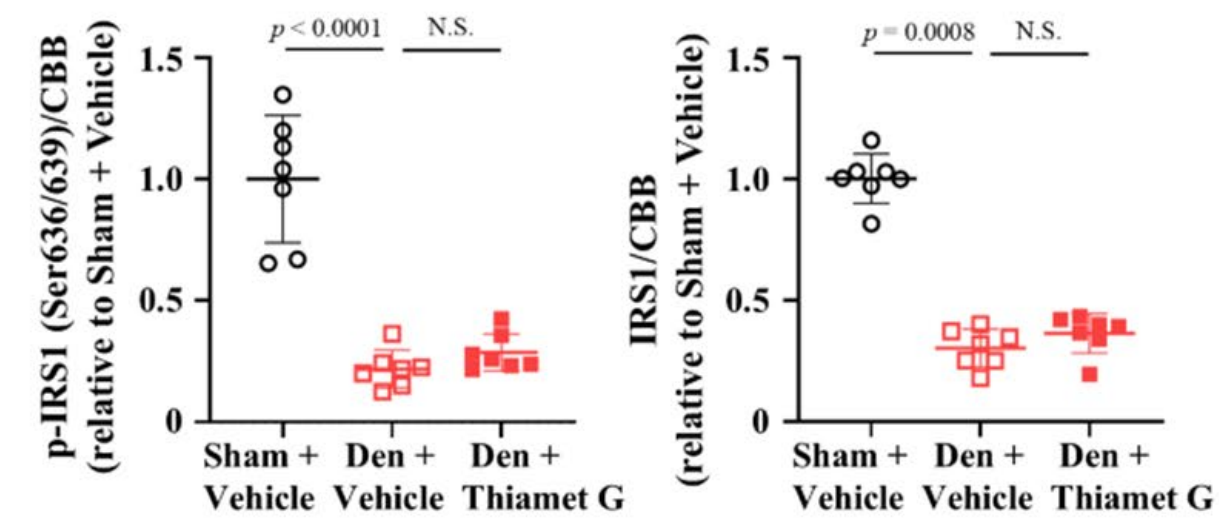

Figure S10

Supplement: Supplementary file 11 — Figure S10: Insulin receptor substrate 1 (IRS1) level is not affected by thiamet G treatment. Representative western blots (left) and summary data (right) of IRS1and p‐IRS1 (Ser636/639) levels in the gastrocnemius muscle of sham + vehicle, denervation (Den) + vehicle and Den + thiamet G (n = 7 in each group). Results were normalized to non‐specific bands of the CBB‐stained gel. Data are shown as the mean ± SD. p values were calculated by one‐way ANOVA followed by the Tukey post hoc test or Kruskal–Wallis test, followed by Dunn's post hoc test. Den, denervation; p‐IRS1, phosphorylated insulin receptor substrate 1; CBB, Coomassie Brilliant Blue; N.S., not significant. [file JCSM-16-e70066-s009.pdf]

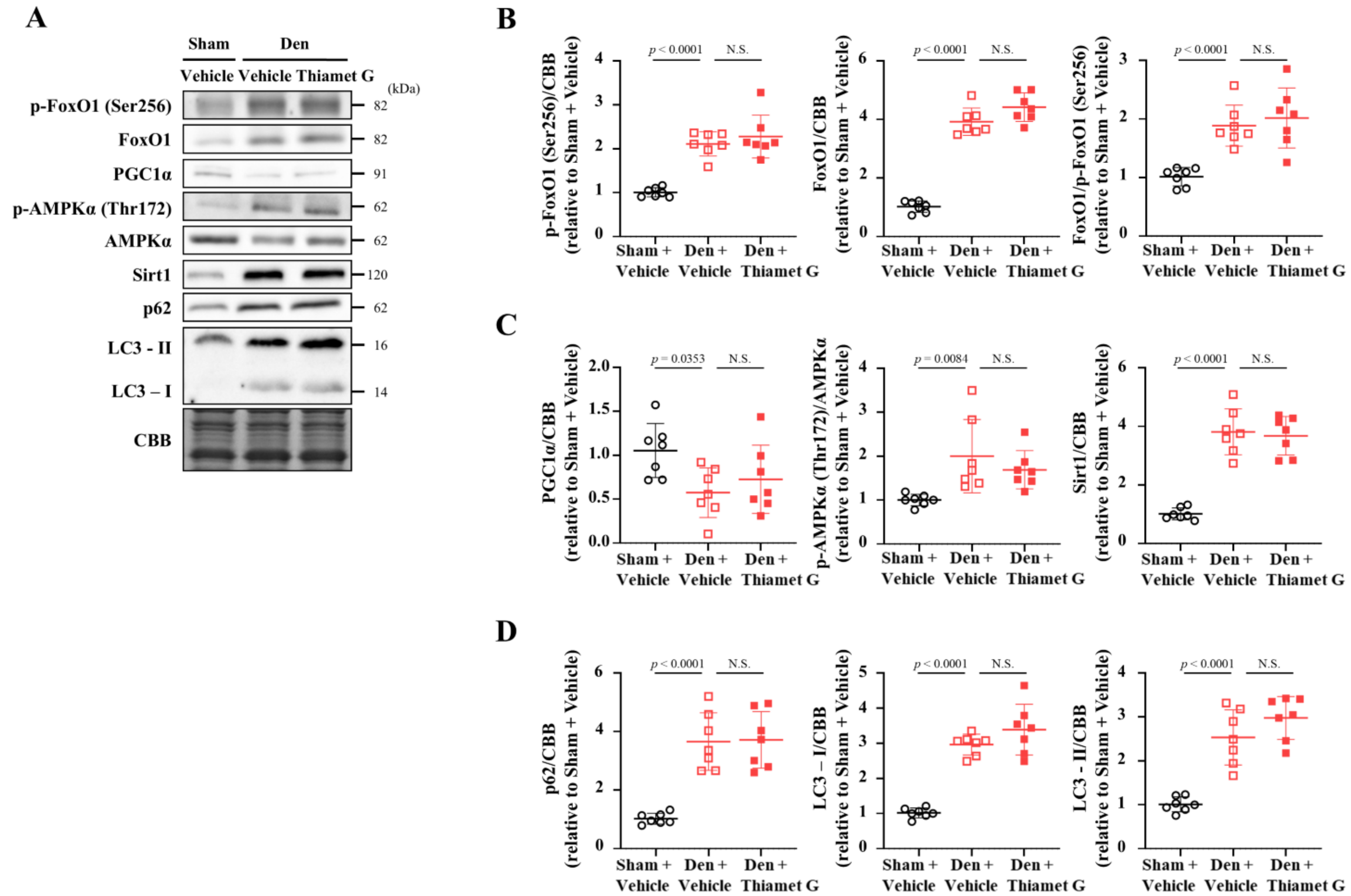

Figure S11

Supplement: Supplementary file 12 — Figure S11: Expression of proteins associated with FoxO1, mitochondrial biogenesis and autophagy. Representative western blots (A) and summary data (B) of p‐FoxO1, FoxO1 and FoxO1/p‐FoxO1, (C) PGC‐1α, p‐AMPKα/AMPKα and Sirt1, (D) p62, LC3‐I and LC3‐II in the gastrocnemius muscle of sham + vehicle (n = 7), denervation (Den) + vehicle (n = 7) and Den + thiamet G (n = 7). The blots were normalized to the non‐specific bands of CBB‐stained gel. Data are shown as the mean ± SD. p values were calculated by one‐way ANOVA, followed by the Tukey post hoc test. CBB, Coomassie Brilliant Blue; Den, denervation; LC3, light chain 3; p‐AMPKα, phosphorylated AMP‐activated protein kinase α; p‐FoxO1, phosphorylated forkhead box O1; PGC‐1α, peroxisome proliferator‐activated receptor γ coactivator‐1 α; Sirt1, sirtuin 1. [file JCSM-16-e70066-s011.pdf]

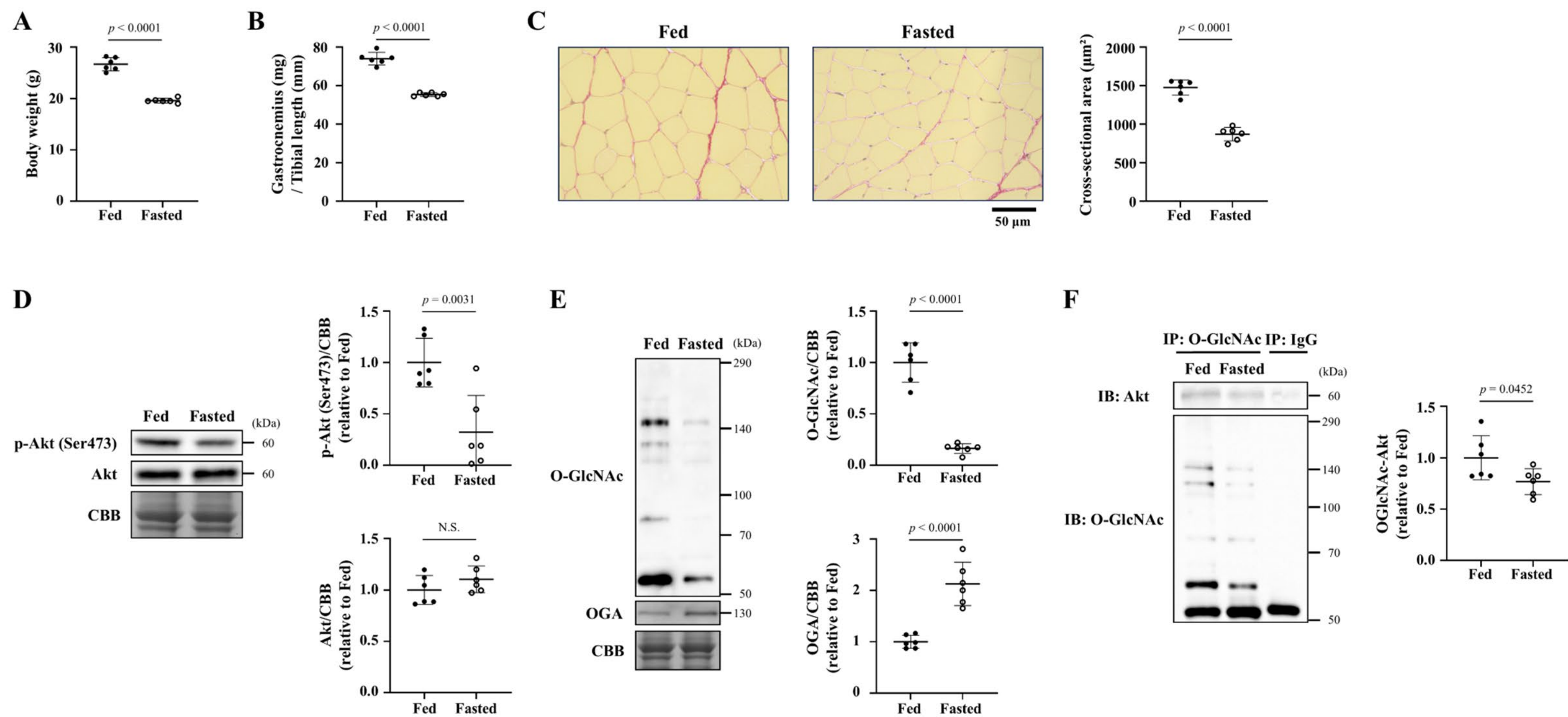

Figure S12

Supplement: Supplementary file 13 — Figure S12: O‐GlcNAcylation of Akt is reduced in fasting‐induced muscle atrophy. Summary data of body weight (A) and gastrocnemius weight/tibial length (B) in fed and fasted mice (n = 6 in each group). (C) Representative high‐magnification photomicrographs (left) and summary data (right) of gastrocnemius tissue sections stained with Picrosirius Red of the two groups (n = 6 in each group). Representative western blots (left) and summary data (right) of p‐Akt (Ser473) and Akt (D) and O‐GlcNAc and OGA (E) levels of the two groups (n = 6 in each group). Results were normalized to non‐specific bands of the CBB‐stained gel. (F) Immunoprecipitation assays using gastrocnemius muscle lysates from the two groups (n = 6 in each group). After immunoprecipitation with control IgG or an O‐GlcNAc antibody, immunoblotting for Akt and O‐GlcNAc was performed. Representative western blots (left) and summary of the data (right) of O‐GlcNAc‐Akt are shown. Data are shown as the mean ± SD. p values were calculated by the unpaired Student t‐test. IB, immunoblotting; IP, immunoprecipitation; NS, not significant; O‐GlcNAc, O‐linked N‐acetylglucosamine; O‐GlcNAc‐Akt, O‐GlcNAcylated Akt; p‐Akt, phosphorylated Akt. [file JCSM-16-e70066-s005.pdf]

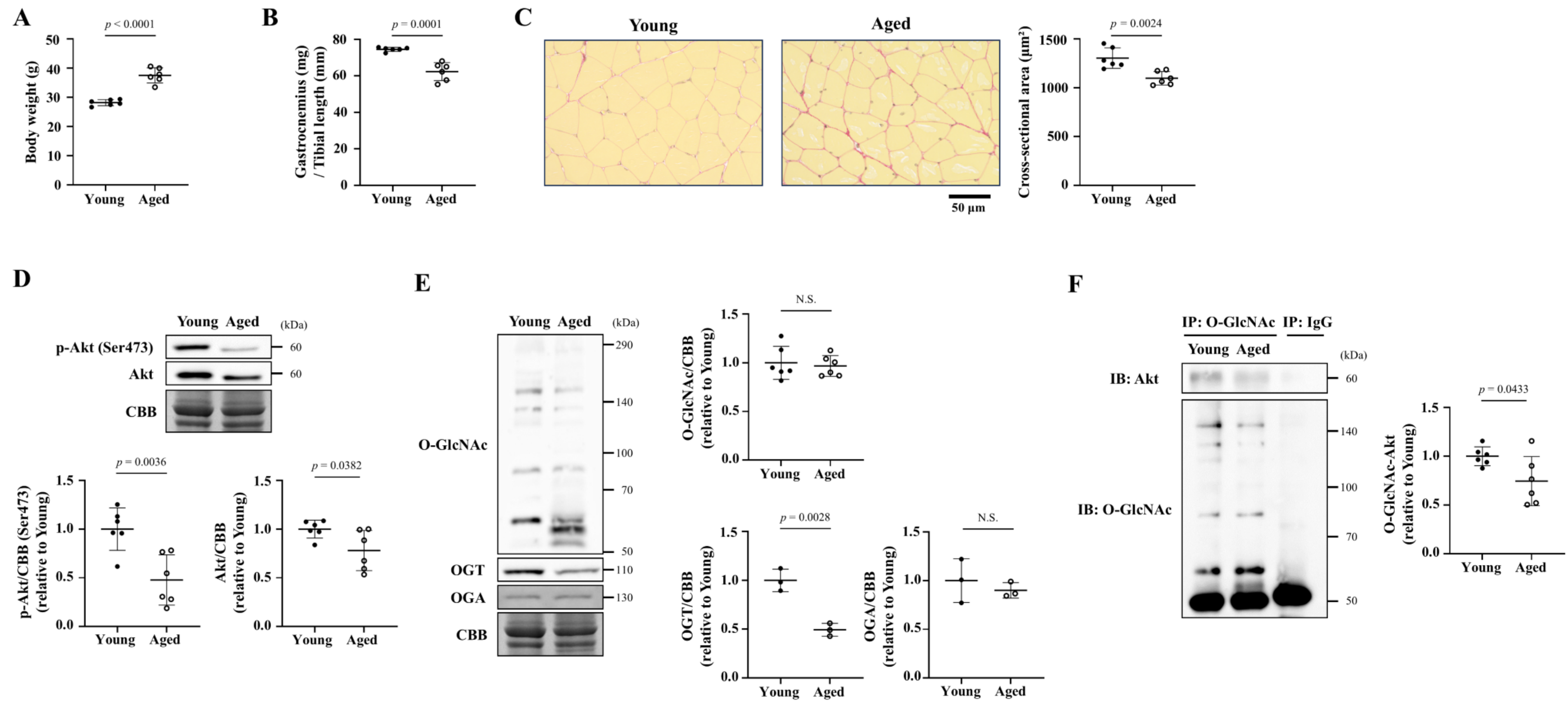

Figure S13

Supplement: Supplementary file 14 — Figure S13: O‐GlcNAcylation of Akt is reduced in age‐associated muscle atrophy. Summary data of body weight (A) and gastrocnemius weight/tibial length (B) in young and aged mice (n = 5 in each group). (C) Representative high‐magnification photomicrographs (left) and summary data (right) of the cross‐sectional area of myocytes in gastrocnemius tissue sections stained with Picrosirius Red of the two groups (n = 6 in each group). Representative western blots (top) and summary data (bottom) of p‐Akt (Ser473) and Akt (D) in the two groups (n = 6 in each group). Representative western blots (left) and summary data (right) of O‐GlcNAc, OGT and OGA (E) levels in the two groups (n = 6, 3, 3 in each group). Results were normalized to non‐specific bands of the CBB‐stained gel. (F) Immunoprecipitation assays using gastrocnemius lysates of in the two groups (n = 6 in each group). After immunoprecipitation with control IgG or an O‐GlcNAcylation antibody, immunoblotting for Akt and O‐GlcNAc was performed. Representative western blots (left) and summary data (right) of O‐GlcNAc‐Akt are shown. Data are shown as the mean ± SD. p values were calculated by the unpaired Student t‐test. IB, immunoblotting; IP, immunoprecipitation; N.S., not significant; O‐GlcNAc, O‐linked N‐acetylglucosamine; O‐GlcNAc‐Akt, O‐GlcNAcylated Akt; p‐Akt, phosphorylated Akt. [file JCSM-16-e70066-s014.pdf]

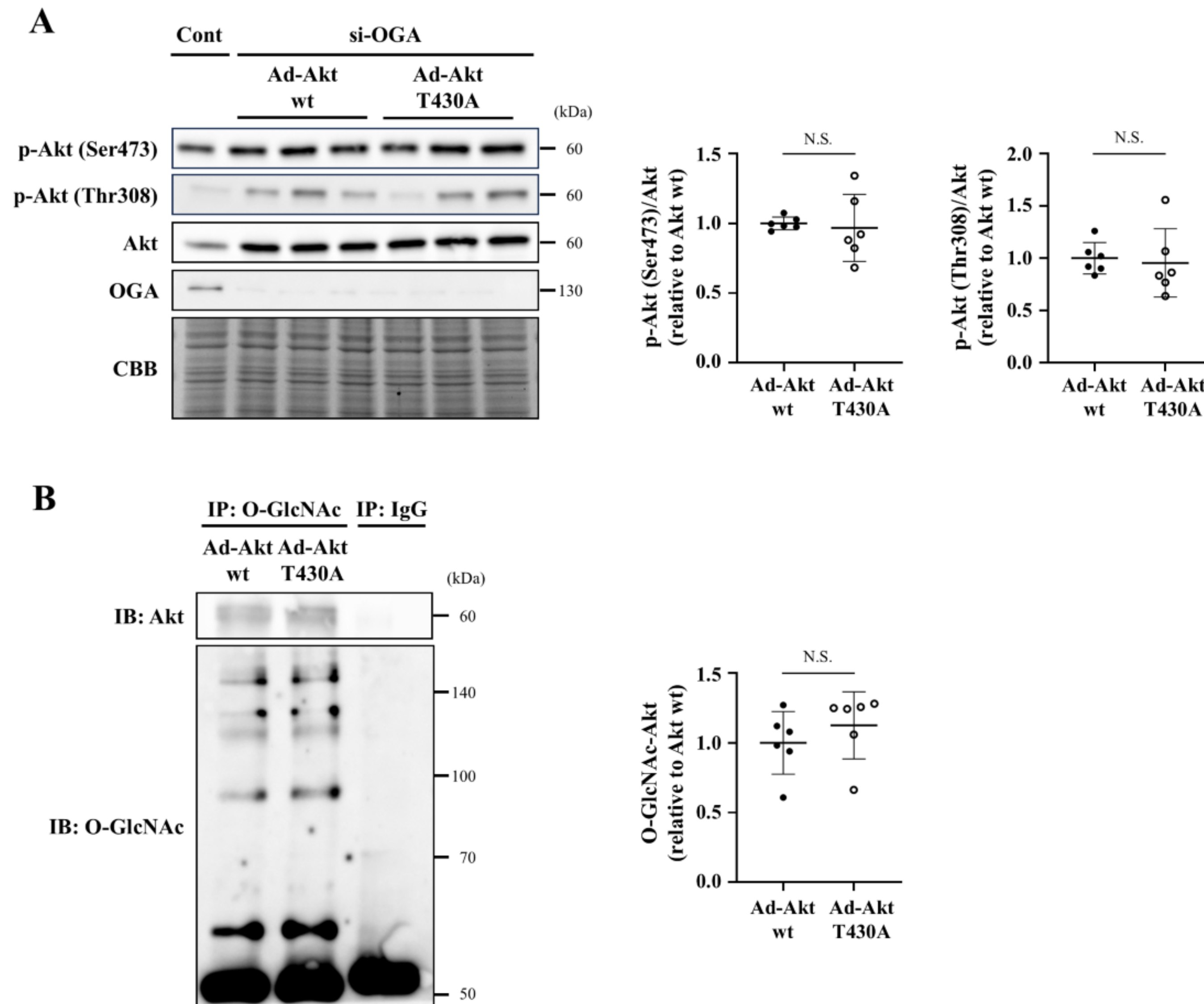

Figure S14

Supplement: Supplementary file 15 — Figure S14: No interaction between phosphorylation at serine 473 and O‐GlcNAcylation of Akt at threonine 430 in C2C12 myotubes. (A) Representative immunoblotting (left) and summary data (right) of p‐Akt (Ser473 and Thr308) and Akt levels in C2C12 myotubes treated with an adenovirus expressing wild‐type Akt (Ad‐Akt wt) or mutant Akt (T430A) (Ad‐Akt T430A), transfected with a small‐interfering RNA against O‐GlcNAcase (si‐OGA) (n = 6 in each group). p‐Akt was normalized to total Akt. Immunoblots in C2C12 myotubes transfected with si‐Scramble were also shown. (B) Immunoprecipitation assays using lysates of C2C12 myotubes in two groups (n = 6 in each group). After immunoprecipitation with control IgG or an O‐GlcNAc antibody, immunoblotting for Akt and O‐GlcNAc was performed. Representative western blots (left) and summary data (right) of O‐GlcNAc‐Akt are shown. Data are shown as the mean ± SD. p values were calculated by the unpaired Student t‐test or Mann–Whitney U test. ad, adenovirus; CBB, Coomassie Brilliant Blue; IB, immunoblotting; IP, immunoprecipitation; NS, not significant; OGA, O‐GlcNAcase; O‐GlcNAc, O‐linked N‐acetylglucosamine; O‐GlcNAc‐Akt, O‐GlcNAcylated Akt; p‐Akt, phosphorylated Akt; siRNA, small interfering RNA; wt, wild type. [file JCSM-16-e70066-s003.pdf]
